# Offline Policy Evaluation and Learning with Harm Constraints

**DOI:** 10.3390/e28101058

**Published:** 2026-09-25

**Authors:** Jile Chaoge, Qinwei Yang, Jingyi Li, Peng Wu

**Affiliations:** 1 School of Mathematics and Statistics, Beijing Technology and Business University, Beijing 102488, China; chogjill@st.btbu.edu.cn (J.C.); qwyang@st.btbu.edu.cn (Q.Y.); 2 School of Statistics, University of International Business and Economics, Beijing 100029, China

**Keywords:** causal inference, individual treatment rules, trustworthy AI

## Abstract

Offline policy learning aims to optimize individualized decisions using historical data. However, conventional methods primarily focus on maximizing expected rewards while neglecting individual-level counterfactual harm—cases where the assigned treatment leads to worse outcomes than the control. This can result in overly aggressive policies that harm some individuals. In this article, we propose a counterfactual harm-aware policy learning framework that effectively balances expected reward with individual-level harm. To quantify harm, we introduce two measures: treatment harm rate (THR), capturing the proportion of individuals harmed, and treatment harm quantity (THQ), capturing the severity of harm, particularly for continuous outcomes. A key challenge is that both THR and THQ depend on the joint distribution of potential outcomes, which is generally unidentifiable, even in randomized controlled trials. To address this, we introduce an additive latent variable model under which we establish the identifiability of THR and THQ and develop the corresponding estimators. These estimators are then used to learn policies under explicit harm constraints. Extensive experiments demonstrate the effectiveness of the proposed approach.

## 1. Introduction

Offline policy learning plays a pivotal role in data-driven, individualized decision-making by leveraging historical data to develop tailored treatment or intervention strategies. Its ability to produce more informed and effective decisions has garnered increasing interest across various domains [1,2,3]. Typical offline policy learning methods primarily aim to maximize expected rewards [4,5]. Beyond reward maximization, trustworthy policies should also adhere to principles such as non-maleficence, justice, and fairness [6,7,8,9,10,11,12].

Mitigating harm is a critical consideration in trustworthy policy learning, particularly in high-stakes domains. For instance, in healthcare, the Hippocratic Oath emphasizes the fundamental principle of prioritizing patient safety and well-being, encapsulated in the maxim “first, do no harm” [13,14,15]. Let $Y^{1}$ and $Y^{0}$ denote the potential outcomes under treatment and control, respectively, and assume that larger outcomes are preferable without loss of generality. Following Kallus [16], a treatment is considered (counterfactually) harmful to an individual if assigning it results in a worse outcome than under control, i.e., $Y^{1}-Y^{0}<0$. The policy harm is then defined as the expected harm incurred when the policy is applied to the entire population. Unfortunately, policy learning approaches that maximize only the expected reward often inadvertently overlook individual-level harm [17,18], which may result in overly aggressive policies that expose some individuals to harm.

In this article, we aim to develop an offline policy learning approach that maximizes the expected reward subject to an explicit constraint on counterfactual harm. A central challenge in this setting lies in evaluating policy harm, which depends on the joint distribution of potential outcomes, a quantity that is generally unidentifiable, even in randomized controlled trials. Recent work [18] tackles this challenge by deriving an upper bound (worst-case) on policy harm and learning policies that maximize expected reward while keeping worst-case policy harm below the given threshold. However, the method proposed by Li et al. [18] leaves two limitations unaddressed. First, it does not address the more complex case of continuous outcomes, where measuring policy-induced harm is more nuanced. Second, it controls policy harm by bounding the worst-case scenario, which may lead to overly conservative policies that significantly compromise expected reward to limit harm. Our proposed method addresses both of these limitations.

For continuous outcomes, we formally discuss statistical measures for quantifying policy harm. Unlike the binary case, it is necessary to consider not only the sign of $Y^{1}-Y^{0}$ but also the magnitude when this difference is negative. For binary outcomes, previous studies [16,18] use the treatment harm rate (THR) to measure the proportion of individuals harmed by the treatment. We propose the concept of treatment harm quantity (THQ), which quantifies the magnitude of harm for continuous outcomes in a more detailed manner. This allows us to capture both the prevalence and severity of harm and thus effectively helps guide policy learning by incorporating THR or THQ (or both) as constraints while optimizing the expected reward.

We next address the identification of THR and THQ. The key condition for identifiability is the additive model for potential outcomes. It posits that the noise variable is additive for potential outcomes. We demonstrate the identifiability of THR and THQ under the additive model, along with the standard strong ignorability assumption [19]. Based on these identifiability results, we propose maximum likelihood and regression-based estimators for THR and THQ. Furthermore, we develop an offline policy learning approach with harm constraints based on the estimated THR and THQ. We also establish the consistency of the estimation error and derive regret bounds for the learned policy.

The main contributions of this article are as follows:

- We formalize the problem of offline policy learning with counterfactual harm constraints for continuous outcomes.
- We establish the identification of THR and THQ for continuous outcomes under an additive latent variable model.
- We develop estimators for THR and THQ and incorporate the estimated harm measures into offline policy learning with harm constraints.
- We conduct extensive experiments to validate the effectiveness of the proposed method.

## 2. Problem Formulation

### 2.1. Notation

Suppose that a simple random sample of *n* units is drawn from a superpopulation P. For each unit, we observe a covariate vector $X_{i}\in X\subseteq R^{m}$, a binary treatment $T_{i}\in T=\left\{0,1\right\}$, where $T_{i}=1$ indicates that the unit received the treatment and $T_{i}=0$ indicates the control, and an outcome $Y_{i}\in Y\subseteq R$. Without loss of generality, we assume that a larger value of $Y_{i}$ is strictly more desirable. We use the widely adopted potential outcome framework [20,21] to define causal effects. Let $Y_{i}^{0}$ and $Y_{i}^{1}$ be the potential outcomes under treatment T=0 and 1, respectively. We maintain the stable unit treatment value assumption, meaning that there is no interference between units and no variation in the form of the treatment. Under this assumption, the observed outcome is $Y_{i}=\left(1-T_{i}\right)Y_{i}^{0}+T_{i}Y_{i}^{1}$, which establishes the connection between observed outcomes and potential outcomes.

The individual treatment effect (ITE) is defined as ${\mathrm{ITE}}_{i}=Y_{i}^{1}-Y_{i}^{0},$ where ${\mathrm{ITE}}_{i}>0$ indicates receiving treatment is beneficial for unit *i*, and ${\mathrm{ITE}}_{i}<0$ indicates that receiving treatment is harmful. Thus, the ITE can serve as the gold standard for guiding individualized decision-making. However, ${\mathrm{ITE}}_{i}$ is generally not identifiable as only one of $Y_{i}^{1}$ or $Y_{i}^{0}$ is observed for each unit, but not both. This is known as the fundamental problem of causal inference [22]. In practice, we typically use the conditional average treatment effect (CATE), defined as

$$\tau \left(x\right)=E\left({\mathrm{ITE}}_{i}|X_{i}=x\right)=E\left(Y_{i}^{1}-Y_{i}^{0}|X_{i}=x\right),$$

to inform tailored decision-making. For an individual with $X_{i}=x$, treatment is recommended if τ(x)>0, and is not recommended otherwise. For ease of presentation, we omit the subscript *i* and use (X,T,Y) to denote a generic unit drawn from a population P.

### 2.2. Formulation

Several studies [16,23,24] have revealed that using CATE to evaluate the effectiveness of a treatment at the individual level is inherently limited and may lead to misleading or even harmful results. Specifically, CATE represents the average treatment effect in the subpopulation where X=x. However, it ignores aspects of individual treatment effect heterogeneity within this subpopulation. For example, in subpopulation X=x, consider a treatment that benefits 70% of individuals but harms 20% (${\mathrm{ITE}}_{i}<0$). Alternatively, there is another treatment that benefits only 40% of individuals but harms no one. In high-stakes domains, it is evident that the latter option should be preferred, even though the former has a higher CATE.

To address this limitation, Shen et al. [25] introduced the treatment harm rate (THR) to measure the proportion of individuals harmed by the treatment. The THR in the subpopulation X=x is defined as

$$\mathrm{THR}\left(x\right)=P\left(Y^{1}-Y^{0}<0|X=x\right).$$

In this work, we further propose the treatment harm quantity (THQ) to quantify the magnitude of harm, as shown in Definition 1.

**Definition** **1** (THQ)**.** *The THQ in the subpopulation X=x is defined as*

$$\begin{array}{c} \mathrm{THQ}\left(x\right)=E[\left(Y^{0}-Y^{1}\right)I\left(Y^{1}-Y^{0}<0\right)|X=x]. \end{array}$$

In the subpopulation of X=x, THR(x) measures the proportion of harmed individuals, while THQ(x) quantifies the extent of harm. For binary outcomes (Y∈{0,1}), since $Y^{1}-Y^{0}=-1$ whenever $Y^{1}-Y^{0}<0$, THR(x) and THQ(x) are equivalent, i.e., THR(x)=THQ(x). In the more general case where assigning a treatment has a cost c(x)⩾0, we may define ${\mathrm{THR}}_{c(x)}\left(x\right)$ and ${\mathrm{THQ}}_{c(x)}\left(x\right)$ as $P\left(Y^{1}-Y^{0}<c\left(x\right)|X=x\right)$ and $E[\left(Y^{0}-Y^{1}+c\left(x\right)\right)I\left(Y^{1}-Y^{0}<c\left(x\right)\right)|X=x]$, respectively, to account for the cost. In the main text, we omit the cost for simplicity, as the main results naturally extend to this setting, see Appendix D for more details.

The THR(x) and THQ(x) provide complementary information about individual-level treatment heterogeneity that is not captured by the CATE, offering critical information for individualized decision-making. Unfortunately, typical policy learning relies on the CATE, aiming to maximize the average reward within covariate-defined subpopulations. As a result, CATE-based policy learning methods may be overly aggressive and potentially harm many individuals.

Our goal is to learn a harm-constrained policy that achieves optimal reward while ensuring that the proportion of harmed individuals or the extent of harm remains within a predefined threshold. Formally, let π:X→[0,1], be a policy that maps individual covariates to treatment probabilities, where π(x) denotes the probability of assigning treatment given X=x. For a given policy π, the policy reward is defined as

$$R\left(\pi \right)=E\left[\pi \left(X\right)Y^{1}+\left(1-\pi \left(X\right)\right)Y^{0}\right],$$

and the THR and THQ induced by this policy are given as

$$\begin{array}{cc} \mathrm{THR}(\pi ) & =E[\mathrm{THR}(X)\pi (X)],\\ \mathrm{THQ}(\pi ) & =E[\mathrm{THQ}(X)\pi (X)], \end{array}$$

which represent the average THR and THQ when applying π to the entire population. Then we aim to learn a policy that solves the following optimization problems:

(1) $$\begin{array}{c} \left\{\begin{array}{c} \max_{\pi \in \Pi }R\left(\pi \right)\\ s.t.\;\mathrm{THR}(\pi )⩽\delta , \end{array}\right.\mathrm{or}\;\left\{\begin{array}{c} \max_{\pi \in \Pi }R\left(\pi \right)\\ s.t.\;\mathrm{THQ}(\pi )⩽\delta , \end{array}\right. \end{array}$$

where Π is a policy class and δ>0 is a pre-specified threshold used to constrain either THR or THQ based on practical considerations.

Unlike typical policy learning methods that focus on maximizing policy reward [26,27], the optimization problem (1) considers both policy reward and potential harms. It helps prevent overly aggressive policies and is particularly relevant for individualized decision-making in high-stakes domains such as health care [15] and public policy [23].

## 3. Identifiability Assumptions

To learn policies under harm constraints, we first need to establish the identifiability of R(π), THR(π) and THQ(π) for a given policy π. Identifiability ensures that these target measures can be expressed in terms of the observed data distribution, thereby enabling consistent estimation and inference in practice. To achieve this, we introduce the commonly used strong ignorability assumption [19].

**Assumption** **1** (Strong Ignorability)**.** *(a) $T⊥\;\;\;⊥(Y^{0},Y^{1})|X$; (b) η<e(x):=P(T=1|X=x)<1−η for some constant η∈(0,1/2), for all x∈X.*

Assumption 1(a) states that the treatment is independent of the potential outcomes conditional on observed covariates. This implies that observed covariates are sufficient to eliminate the confounding bias between treatment and outcome [28]. Assumption 1(b) ensures that each individual has a strictly positive probability of receiving each treatment. Assumption 1 is widely adopted in the causal inference literature [19,29]. Under Assumption 1, for a given policy π, R(π) is identified as follows,

R(π)=E[π(X)μ(X,1)+(1−π(X))μ(X,0)],

where μ(X,t)=E[Y∣X,T=t] is the outcome regression function for t=0,1.

However, Assumption 1 is not sufficient to identify THR(x) and THQ(x), as their definitions involve the joint distribution $P(Y^{0},Y^{1}|X=x)$. A commonly used assumption is conditional independence between potential outcomes [30], that is,

$$Y^{0}⊥\;\;\;⊥Y^{1}|X.$$

Under this assumption, the joint distribution can be factorized as $P\left(Y^{0}|X\right)P\left(Y^{1}|X\right)$. However, this assumption is often too stringent in practice and may not hold in many real-world applications. Rather than decomposing the joint distribution $P(Y^{0},Y^{1}|X)$ into marginal components, we propose a novel identifiability condition based on an additive latent variable model, which ensures the identifiability of THR(x) and THQ(x).

**Assumption** **2** (Additive Model)**.** *Suppose that X,T, and Y satisfy an additive model:*

(2) $$\begin{array}{c} \left\{\begin{array}{c} Y=\mu (X,T)+\sigma (X,T)U\\ U|X∼N\left(0,1\right),\;\sigma \left(X,T\right)>0, \end{array}\right. \end{array}$$

*where T∈{0,1} is the treatment and U is the unmeasured noise variable.*

We first give two remarks on Assumption 2.

**Remark** **1** (Mean and variance of potential outcomes)**.** *In model *(2)*, μ(X,t) and σ(X,t) represent the conditional mean and standard deviation of $Y^{t}$, respectively. Under Assumption 1, $E\left(Y^{t}|X\right)=\mu \left(X,t\right)$ and $\mathrm{Var}\left(Y^{t}|X\right)=\sigma^{2}\left(X,t\right)$, so both quantities are identifiable.*

**Remark** **2** (Interpretation on *U* and Plausibility of Assumption 2)**.** *Under Assumption 2, the potential outcomes satisfy $Y^{t}=\mu \left(X,t\right)+\sigma \left(X,t\right)U$, t∈{0,1}, so that the same latent variable U affects the outcome under both treatment conditions. The variable U can be interpreted as an unobserved individual-specific characteristic that remains stable across treatment conditions.*

Assumption 2 is plausible in many biomedical and social science applications where an individual’s latent/intrinsic characteristic *U* influences the outcome regardless of treatment assignment. For example, in clinical studies, *U* may represent an individual’s underlying physiological resilience or genetic susceptibility, which influences outcomes regardless of treatment exposure. Similarly, in educational interventions, *U* may capture a student’s innate learning ability or long-term motivation, which affects performance under either instructional strategy. In such settings, treatment modifies the expected outcome, while the latent individual heterogeneity *U* remains shared across treatment conditions, making Assumption 2 a reasonable approximation. Nevertheless, Assumption 2 may not be appropriate in several applications. For example, it may fail when treatment introduces new individual-specific mechanisms that do not exist under control, so that different latent factors govern the two potential outcomes. In addition, the assumption relies on the conditional normality of the latent variable, which may be violated when the unobserved heterogeneity exhibits strong skewness or heavy tails.

Assumption 2 imposes substantive restrictions on the cross-world dependence between $Y^{0}$ and $Y^{1}$ [24] through the shared latent variable *U*. While this assumption is plausible in settings where a stable individual characteristic influences outcomes regardless of treatment (e.g., genetic predisposition or physiological resilience), it may be violated when treatment introduces new mechanisms that are absent under control.

Notably, the additive model (2) is highly flexible, as it leaves the functional forms of μ(X,T) and σ(X,T) unspecified. Furthermore, we show that the additive model (2) remains valid even if *U* depends on *X* (or *U* is related to *X*). Below, we provide an example to illustrate it.

**Example** **1** (*U* depends on *X* under the additive model)**.** *Suppose that the true model is*

(3) $$\begin{array}{c} \left\{\begin{array}{c} Y=\mu (X,T)+\sigma (X,T)U,\;\sigma (X,T)>0\\ U|X∼N\left(M\left(X\right),D^{2}\left(X\right)\right),\;D\left(X\right)>0. \end{array}\right. \end{array}$$

*In model *(3)*, the condition “U∣X∼N(0,1)” is violated. Nevertheless, we could write U as $M\left(X\right)+D\left(X\right)\tilde{U}$ (i.e., let $\tilde{U}=\left(U-M\left(X\right)\right)/D\left(X\right)$), where $\tilde{U}|X∼N\left(0,1\right)$. Let $\tilde{\mu }\left(X,T\right)=\mu \left(X,T\right)+\sigma \left(X,T\right)M\left(X\right)$ and $\tilde{\sigma }\left(X,T\right)=\sigma \left(X,T\right)D\left(X\right)$. Then model *(3)* can be reformulated as*

$$\begin{array}{c} \left\{\begin{array}{c} Y=\tilde{\mu }\left(X,T\right)+\tilde{\sigma }\left(X,T\right)\tilde{U}\\ \tilde{U}|X∼N\left(0,1\right),\;\tilde{\sigma }\left(X,T\right)>0. \end{array}\right. \end{array}$$

*Therefore, model *(3)* reduces to the form of model *(2)*.*

Despite the flexibility of Assumption 2, it is fundamentally untestable from the observed data because it concerns the joint distribution of the two potential outcomes, only one of which is observed for each individual. Consequently, the plausibility of this assumption must be justified based on substantive scientific knowledge rather than empirical goodness-of-fit alone. Under Assumptions 1 and 2, we have the following identifiability results.

**Theorem** **1** (Identification of THR(x) and THQ(x))**.** *Under Assumptions 1 and 2, THR(x) and THQ(x) are identifiable,*

*(i)*  *When σ(x,1)≠σ(x,0), the identifiability formulas are as follows:*
  $$\begin{array}{cc} \mathrm{THR}(x)= & \Phi \left(\frac{\mu (x,0)-\mu (x,1)}{\left|\sigma (x,1)-\sigma (x,0)\right|}\right),\\ \mathrm{THQ}(x)= & \left(\mu (x,0)-\mu (x,1)\right)\Phi \left(\frac{\mu (x,0)-\mu (x,1)}{\left|\sigma (x,1)-\sigma (x,0)\right|}\right)\\ & +\frac{\left|\sigma (x,1)-\sigma (x,0)\right|}{\sqrt{2\pi }}\exp\left\{-\frac{{\left(\mu \left(x,1\right)-\mu \left(x,0\right)\right)}^{2}}{2{\left(\sigma (x,1)-\sigma (x,0)\right)}^{2}}\right\}, \end{array}$$
  *where Φ(·) denotes the cumulative distribution function of the standard normal distribution.*
*(ii)*  *When σ(x,1)=σ(x,0), the identifiability formulas are as follows:*
  $$\begin{array}{cc} \; & \mathrm{THR}(x)=I(\mu (x,1)-\mu (x,0)<0),\\ \; & \mathrm{THQ}(x)=(\mu (x,0)-\mu (x,1))I(\mu (x,1)-\mu (x,0)<0). \end{array}$$

Theorem 1 establishes the identifiability of THR(x) and THQ(x) under Assumptions 1 and 2. Theorem 1(ii) is a degenerate case of Theorem 1(i), corresponding to the limiting form of Theorem 1(i) as |σ(x,1)−σ(x,0)| goes to zero. In addition, noting that THR(π)=E[THR(X)π(X)] and THQ(π)=E[THQ(X)π(X)], Theorem 1 implies the identifiability of THR(π) and THQ(π) for any given policy π.

## 4. Offline Policy Learning with Harm Constraints

Building on the identifiability results in Section 3, we estimate THR(π) and THQ(π) for a given policy π, and use these estimates to learn policies under harm constraints.

**Estimation of THR(π) and THQ(π).** The proposed estimation procedures are given as follows.

- **Step 1:** estimate μ(x,t)=E[Y∣X=x,T=t] for t=0,1. Let $\hat{\mu }\left(x,t\right)$ denote its estimator.
- **Step 2:** estimate σ(x,t), and let $\hat{\sigma }\left(x,t\right)$ be its estimator.
- **Step 3:** estimate THR(π) and THQ(π) using the plug-in method. The estimator of THR(π) is given by $\hat{\mathrm{THR}}\left(\pi \right)=n^{-1}\sum_{i=1}^{n}\hat{\mathrm{THR}}\left(X_{i}\right)\pi \left(X_{i}\right)$, where
  $$\hat{\mathrm{THR}}\left(x\right)=\Phi \left(\frac{\hat{\mu }\left(x,0\right)-\hat{\mu }\left(x,1\right))}{\left|\hat{\sigma }\left(x,1\right)-\hat{\sigma }\left(x,0\right)\right|}\right).$$
  Similarly, the estimator of THQ(π) is given by $n^{-1}\sum_{i=1}^{n}\hat{\mathrm{THQ}}\left(X_{i}\right)\cdot \pi \left(X_{i}\right),$ where
  $$\begin{array}{cc} \hat{\mathrm{THQ}}\left(x\right)= & \left(\hat{\mu }\left(x,0\right)-\hat{\mu }\left(x,1\right)\right)\Phi \left(\frac{\hat{\mu }\left(x,0\right)-\hat{\mu }\left(x,1\right)}{\left|\hat{\sigma }\left(x,1\right)-\hat{\sigma }\left(x,0\right)\right|}\right)\\ \; & +\frac{\left|\hat{\sigma }\left(x,1\right)-\hat{\sigma }\left(x,0\right)\right|}{\sqrt{2\pi }}\exp\left\{-\frac{{\left(\hat{\mu }\left(x,1\right)-\hat{\mu }\left(x,0\right)\right)}^{2}}{2{\left(\hat{\sigma }\left(x,1\right)-\hat{\sigma }\left(x,0\right)\right)}^{2}}\right\}. \end{array}$$

In Step 1, we could estimate μ(x,t) by directly regressing *Y* on *X* using data with T=t. In Step 2, note that under Assumptions 1 and 2 (see Appendix B),

$$\begin{array}{cc} \sigma^{2}\left(x,t\right)= & E[{\left\{Y^{t}-E\left(Y^{t}|X\right)\right\}}^{2}|X=x]\\ = & E[{\left(Y-\mu \left(X,t\right)\right)}^{2}|X=x,T=t]. \end{array}$$

Thus, we can estimate $\sigma^{2}\left(x,t\right)$ by regressing ${\left(Y-\hat{\mu }\left(X,t\right)\right)}^{2}$ on *X* using the data with T=t. In addition, we could estimate $\sigma^{2}\left(x,t\right)$ using the maximum likelihood estimation method. Specifically, assume that σ(x,t) is parameterized as $\sigma (x,t;\beta_{t})$, where $\beta_{t}$ is the parameter. Given $\hat{\mu }\left(x,t\right)$, the log-likelihood of $\left(\beta_{0},\beta_{1}\right)$ (see Appendix B) is given by

$$\begin{array}{cc} l(\beta_{0},\beta_{1})= & -\frac{n}{2}\log\left(2\pi \right)-\sum_{i=1}^{n}T_{i}\left\{\log\sigma^{2}\left(X_{i},1,\beta_{1}\right)+\frac{1}{2\sigma^{2}\left(X_{i},1,\beta_{1}\right)}{\left(Y_{i}-\hat{\mu }\left(X_{i},1\right)\right)}^{2}\right\}\\ \; & -\sum_{i=1}^{n}\left(1-T_{i}\right)\left\{\log\sigma^{2}\left(X_{i},0,\beta_{0}\right)+\frac{{\left(Y_{i}-\hat{\mu }\left(X_{i},0\right)\right)}^{2}}{2\sigma^{2}\left(X_{i},0,\beta_{0}\right)}\right\}. \end{array}$$

We can then obtain the estimates of $\left(\beta_{0},\beta_{1}\right)$ by maximizing $l(\beta_{0},\beta_{1})$.

**Policy Learning with Harm Constraints.** Based on $\hat{\mathrm{THR}}\left(\pi \right)$ and $\hat{\mathrm{THQ}}\left(\pi \right)$, we learn policies that maximize the expected reward subject to constraints on the harm rate or harm quantity.

For instance, to control the harm rate, the target policy $\pi^{*}$ is the solution of $\max_{\pi \in \Pi }R\left(\pi \right)\;s.t.\;\mathrm{THR}\left(\pi \right)⩽\delta$. We define ${\hat{\pi }}^{*}$ as the solution to the following optimization problem (see Appendix B for more details)

$$\begin{array}{c} \left\{\begin{array}{c} \max_{\pi \in \Pi }\hat{R}\left(\pi \right)\\ s.t.\;\hat{\mathrm{THR}}\left(\pi \right)⩽\delta , \end{array}\right. \end{array}$$

where $\hat{R}\left(\pi \right)$ is the standard doubly robust estimator [31,32], given by

$$\begin{array}{cc} \hat{R}\left(\pi \right)= & \frac{1}{n}\sum_{i=1}^{n}[\left(\frac{T_{i}\left(Y_{i}-\hat{\mu }\left(X_{i},1\right)\right)}{\hat{e}\left(X_{i}\right)}+\hat{\mu }\left(X_{i},1\right)\right)\pi \left(X_{i}\right)\\ \; & +\left(\frac{\left(1-T_{i}\right)\left(Y_{i}-\hat{\mu }\left(X_{i},0\right)\right)}{1-\hat{e}\left(X_{i}\right)}+\hat{\mu }\left(X_{i},0\right)\right)\left(1-\pi \left(X_{i}\right)\right)], \end{array}$$

where $\hat{e}\left(x\right)$ is the estimate of e(x):=P(T=1∣X=x). Here, the threshold δ is imposed on the estimated harm rate $\hat{\mathrm{THR}}\left(\pi \right)$. Therefore, satisfaction of the estimated constraint does not necessarily guarantee exact finite-sample satisfaction of the constraint THR(π)⩽δ.

Next, we study $R\left(\pi^{*}\right)-R\left({\hat{\pi }}^{*}\right)$ and $R\left(\pi^{*}\right)-\hat{R}\left({\hat{\pi }}^{*}\right)$, which represent the regret of the learned policy and the estimation error in its reward, respectively.

**Theorem** **2** (Consistency of Estimation Error and Regret Bound)**.** *Suppose that *Π* is a P-G-C class, $\hat{\mu }\left(x,t\right)$, $\hat{\sigma }\left(x,t\right)$, and $\hat{e}\left(x\right)$ are uniformly consistent estimators of μ(x,t), σ(x,t), and e(x) for t=0,1, respectively, and aπ∈Π for any π∈Π and 0<a<1, then we have,*

*(a)*  $\hat{R}\left({\hat{\pi }}^{*}\right)-R\left(\pi^{*}\right)\overset{P}{\to }0$.
*(b)*  $R\left({\hat{\pi }}^{*}\right)-R\left(\pi^{*}\right)\overset{P}{\to }0$.

Theorem 2 indicates that if the policy class Π is a P-Glivenko–Cantelli (P-G-C) class. Let $X_{1},\ldots ,X_{n}$ be an i.i.d. sample from a population P. A function class F is P-G-C class if $\sup_{f\in F}\left|n^{-1}\sum_{i=1}^{n}f\left(X_{i}\right)-E\left(f\left(X_{i}\right)\right)\right|\overset{a.s.}{\to }0$ [33]. Most commonly used function classes belong to the P-G-C class [34], then under mild conditions, both $\hat{R}\left({\hat{\pi }}^{*}\right)$ and $R({\hat{\pi }}^{*})$ are consistent estimators of $R(\pi^{*})$. If Assumption 2 is violated, the identifiability results in Theorem 1 and the asymptotic results in Theorem 2 no longer hold, and the plug-in estimators for THR and THQ may be biased. In particular, misspecification of the cross-world dependence structure can lead to systematic under- or over-estimation of the harm measures, potentially resulting in policies that fail to satisfy the intended harm constraints. We investigate the sensitivity of our method to violations of the additive structure in Section 5.2 (alternative dependence between potential outcomes), demonstrating that the proposed approach maintains reasonable performance under moderate departures from the assumed model.

## 5. Experiments

We address the following research questions to assess the performance and robustness of the proposed methods:

- **RQ1:** How does the proposed method perform under different harm rate thresholds;
- **RQ2:** How do the two different approaches for estimating σ(x,t) perform;
- **RQ3:** How does the proposed method perform across varying SNR levels.
- **RQ4:** How robust is the proposed method to misspecification in experimental settings, including non-normal noise, alternative dependence structures between potential outcomes, misspecified variance structures and nuisance models?

### 5.1. Experimental Setup

**Datasets and Implementation Details.** We perform experiments using two commonly used benchmark datasets: the semi-synthetic Ihdp, and the real-world Twins, to evaluate the performance of our proposed method. The Ihdp dataset [35] was constructed to investigate the impact of specialist home visits on future cognitive test scores. It includes 672 units (123 treated and 549 controls) and 25 covariates related to the children and their mothers. The Twins dataset is derived from the twin-birth records studied by Almond et al. [36]. The original data come from the U.S. Linked Birth–Infant Death files and focus on twin births in the United States, with the main analysis in the original paper based on births from 1989 to 1991. The dataset contains 5271 units (3016 treated and 2255 control) and 38 covariates describing infant and maternal characteristics.

Inspired by the previous studies [11,18,37], we generate potential outcomes based on both the observed covariates and an unobserved latent variable using the following data-generating mechanisms: $Y_{i}^{0}∼w_{0}X_{i}+\left(\alpha_{0}+\beta_{0}\left|X_{i1}\right|\right)U_{i}$ and $Y_{i}^{1}∼w_{1}X_{i}+\left(\alpha_{1}+\beta_{1}\left|X_{i1}\right|\right)U_{i}$, where $X_{i1}$ represents the first element of the vector $X_{i}$, $w_{0}∼N_{\left[-1,1\right]}\left(0,1\right)$ follows a truncated normal distribution, $w_{1}∼\mathrm{Unif}\left(-1,1\right)$ follows a uniform distribution, and $U_{i}$ is drawn from a standard normal distribution. We set $\alpha_{0}=0.1,\;\alpha_{1}=0.6$ for Ihdp, and $\alpha_{0}=1,\;\alpha_{1}=2$ for Twins. The parameters $\beta_{0}$ and $\beta_{1}$ represent the signal-to-noise ratios (SNR), which can vary across different settings and scenarios. For **RQ1** and **RQ2**, we choose $\beta_{0}=\beta_{1}=0.5$, while in **RQ3**, we vary the SNR values to evaluate the overall robustness of our methods. Detailed implementation procedures are provided in Appendix F.

**Baselines.** We compare the following methods:

- **Naive method:** π(X)≡T. The treatment assignment follows the historical interventions without any modification. This serves as a simple baseline for comparison.
- **CATE-based method**: A method that maximizes only the expected reward, without considering any constraint on the counterfactual harm rate or quantity.
- **Proposed methods:** Our methods optimize expected reward subject to predefined constraints on the counterfactual harm rate, harm quantity, or both.

**Evaluation Metrics:** We report the following four metrics: the change in welfare, the policy rewards, the harm rate, and the harm quantity.

- **Change in welfare:** The change in welfare is defined as $\Delta \mathrm{Welfare}\left(\hat{\pi }\right)=\sum_{i=1}^{n}\left[Y_{i}^{1}-Y_{i}^{0}\right]\hat{\pi }\left(X_{i}\right)$, which quantifies the overall change in welfare induced by the learned policy relative to the null policy π(x)≡0.
- **Reward:** The reward of the learned policy $\hat{\pi }\left(X\right)$ is defined as $R\left(\hat{\pi }\right)=\sum_{i=1}^{n}[\hat{\pi }\left(X_{i}\right)\left(Y_{i}^{1}+\left(1-\hat{\pi }\left(X_{i}\right)\right)Y_{i}^{0}\right]$ which quantifies the overall reward of the policy.
- **True Harm Rate:** The harm rate is defined as $\mathrm{HarmRate}\left(\hat{\pi }\right)=n^{-1}\sum_{i=1}^{n}I\left\{Y_{i}^{1}-Y_{i}^{0}<0\right\}\cdot \hat{\pi }\left(X_{i}\right)$, which measures the rate of harm.
- **True Harm Quantity:** The true harm quantity is defined as $\mathrm{HarmQuantity}\left(\hat{\pi }\right)=n^{-1}\sum_{i=1}^{n}\left(Y_{i}^{0}-Y_{i}^{1}\right)I\left\{Y_{i}^{1}-Y_{i}^{0}<0\right\}\cdot \hat{\pi }\left(X_{i}\right)$, which measures the quantity of harm caused by the learned policy.

### 5.2. Results

**Performance of the proposed methods under different harm rate levels (RQ1).** We evaluate how well our method performs by comparing it against naive and CATE-based baselines across varying harm rate thresholds δ. The results for the Ihdp and Twins datasets are shown in Table 1. Compared with the naive policy, the proposed methods yield greater gains in welfare and reward while substantially reducing both the harm rate and harm quantity. In comparison with the unconstrained CATE-based policy, the proposed methods achieve substantial reductions in harm rate and harm quantity, with a moderate sacrifice in welfare improvement and reward. These results demonstrate the trade-off between reward maximization and harm control.

We also observe that as the harm rate threshold varies, our method consistently strikes a good balance between maximizing reward and minimizing harm. It highlights the robustness of our method in optimizing the policy under different harm constraints. The Naive policy assigns treatment to all individuals and does not involve model fitting or random initialization. Therefore, under a fixed evaluation dataset, its performance is deterministic across trials, leading to zero standard deviation.

Notably, the pre-specified threshold δ is enforced on the estimated $\hat{\mathrm{THR}}\left(\pi \right)$ during policy learning, while the harm rates reported in Table 1 are computed using the ground-truth potential outcomes available in the simulation studies. Consequently, satisfying the estimated constraint does not imply exact finite-sample satisfaction of the corresponding true harm constraint. The exceedances, such as 0.050 under δ=0.03 for Ihdp and 0.0458 under δ=0.02 for Twins, are attributable to finite-sample estimation uncertainty and the resulting gap between estimated constraint control and realized harm rates. Results for policies with harm quantity constraints are reported in Appendix E.

**Performance of the two estimation approaches (RQ2).** The results obtained under the MLE-based and regression-based estimation methods for σ(x,t) are presented in Figure 1 and Figure 2, respectively. From them, one can see that the two estimation approaches perform similarly in terms of all metrics. Similarly, compared with the CATE-based method, the two proposed methods attain lower gains in welfare and reward but achieve a lower harm rate.

Meanwhile, our method consistently reduces the harm rate and harm quantity, highlighting its advantage in managing potential harm in practice.

**Effects of varying SNR levels (RQ3).** We conduct experiments with different SNR levels $\beta_{0},\beta_{1}$ on the Ihdp and Twins datasets. Specifically, we set $\beta_{0}=\beta_{1}$ varying from 0.2 to 0.8. The results for four metrics are shown in Figure 3 and Figure 4. Across different values of $\beta_{0}$ and $\beta_{1}$, the proposed method exhibits stable performance, demonstrating its robustness to SNR levels.

**Robustness Analysis (RQ4).** To further assess the robustness of the proposed method, we conduct additional experiments under various forms of misspecification. Specifically, we consider the following settings.

1. **Non-normal noise.** To test robustness against non-Gaussian latent noise, we replace the standard normal noise by either standardized uniform noise $U∼\mathrm{Unif}(-\sqrt{3},\sqrt{3})$ or centered exponential noise U∼Exp(1)−1, with the threshold parameter δ=0.03. As shown in Table 2, the rate-constrained policy consistently achieves high welfare and reward while maintaining effective harm control, suggesting a favorable balance between utility improvement and harm reduction.
2. **Alternative dependence between potential outcomes.** We vary the dependence between the latent components of the two potential outcomes. Specifically, we set
  $$U_{i1}=\rho U_{i0}+\sqrt{1-\rho^{2}}\epsilon_{i},$$
  with independent $U_{i0}$ and $\epsilon_{i}$. We evaluate ρ∈{0.2,0.8}, with the threshold parameter δ=0.03. Table 3 shows that our method maintains stable performance under varying dependence structures between potential outcomes, including both weak and strong dependence scenarios, demonstrating its robustness.
3. **Nonlinear variance structures.** To assess robustness to variance-model misspecification, we replace the original linear variance scale based on $|X_{i1}|$ with two nonlinear covariate-dependent variance structures:
  $$\sigma_{a}\left(X_{i}\right)=\alpha_{a}+\beta_{a}X_{i1}^{2},\;\sigma_{a}\left(X_{i}\right)=\alpha_{a}+\beta_{a}\left|X_{i1}\right|+0.2X_{i2}^{2}.$$
  These two nonlinear variance structures are used to generate the data for both datasets, whereas the method is implemented using the linear specification with the threshold parameter δ=0.03. The results in Table 4 demonstrate that the proposed method remains robust under nonlinear heteroskedasticity. Across both datasets and variance specifications, the rate-constrained policy consistently reduces harm rate and harm quantity relative to the CATE-based policy, while achieving comparable levels of welfare and reward.
4. **Nuisance-model misspecification.** Finally, we evaluate nuisance-model misspecification by generating outcomes from a nonlinear conditional mean that is not covered by the main linear outcome template. The main nuisance specification assumes a linear conditional mean $w_{a}^{⊤}X_{i}$ with the variance scale $\sigma_{a}\left(X_{i}\right)=\alpha_{a}+\beta_{a}\left|X_{i1}\right|$. In this experiment, the true potential outcomes are generated as
  $$Y_{i}^{0}=w_{0}^{⊤}X_{i}+h_{0}\left(X_{i}\right)+\sigma_{0}\left(X_{i}\right)U_{i0},\;Y_{i}^{1}=w_{1}^{⊤}X_{i}+h_{1}\left(X_{i}\right)+\sigma_{1}\left(X_{i}\right)U_{i1},$$
  where $h_{0}\left(X_{i}\right)=0.5\sin\left(X_{i1}X_{i2}\right)+0.25X_{i3}^{2},\;h_{1}\left(X_{i}\right)=0.5\cos\left(X_{i1}X_{i2}\right)-0.25X_{i2}X_{i3}+0.15X_{i1}^{2}.$ We keep $\sigma_{a}\left(X_{i}\right)=\alpha_{a}+\beta_{a}\left|X_{i1}\right|$, $\beta_{0}=\beta_{1}=0.5$, and set $U_{i1}=0.5U_{i0}+\sqrt{1-0.5^{2}}\epsilon_{i}$. Thus, the nuisance model is misspecified because the true outcome regression contains nonlinear and interaction terms while the policy-learning and harm-estimation pipeline is kept unchanged. Table 5 shows that the rate-constrained policy achieves performance comparable to that under the correctly specified outcome model (Table 1), demonstrating its robustness.

To further assess harm-constraint calibration, we conduct additional IHDP experiments with three nonlinear scale mechanisms under correctly specified and misspecified scale models. We evaluate the frequency and magnitude of true THR exceedances above the prescribed threshold and compare estimated THR with true THR across repeated runs. Full experimental details and results are provided in Appendix E.

## 6. Related Work

**Trustworthy policy learning.** Policy learning aims to identify the individuals who should receive treatment based on covariates [27]. Various methods have been developed to learn policies by maximizing the expected reward, such as regression-based approaches [26], reweighting methods [38], and doubly robust techniques [39]. Trustworthy policy learning involves more than just maximizing reward, it also requires upholding principles such as beneficence, non-maleficence, autonomy, justice, and applicability [7,8,9,11]. A variety of counterfactual-based metrics have been proposed to quantify these trust metrics [40,41,42,43].

Recently, Li et al. [18] proposed a policy learning framework that maximizes expected reward subject to a constraint on the harm rate. Building upon this work, Yang et al. [44] developed semiparametric efficient estimators for the policy reward and harm, enabling efficient learning of the optimal policy. However, their work does not establish the identifiability of the harm rate and instead relying on its upper bound (the worst-case) to control the harm rate. We extend this work by introducing THQ and establishing the identification of both THR and THQ under an additive latent variable model. We further develop two methods for estimating these quantities and incorporate the resulting estimators into offline policy learning with harm constraints.

**Treatment harm rate estimation.** Shen et al. [25] introduced the treatment harm rate (THR) to quantify the harm rate for binary outcomes. Subsequently, several works discussed the identifiability of THR [25,45,46] under conditional independence between potential outcomes; however, this assumption is often too stringent in practice. Instead of focusing on identifiability, several studies explored tight bounds on THR [16,18,24,47,48,49]. Recently, for binary and categorical outcomes, Wu and Mao [50] established nonparametric identification of the joint distribution under binary treatment by combining multiple experimental studies, and Shahn and Madigan [51] subsequently extended this framework. In addition, several studies have investigated treatment harm within the structural causal model framework, providing alternative perspectives on the characterization of counterfactual harm [12,52]. More broadly, existing approaches to identifying the joint distribution of potential outcomes can be grouped into several categories: conditional independence approaches [45] assume $Y^{0}⊥\;\;\;⊥Y^{1}|X$, but this is often too stringent; bounds-based or partial identification approaches [16,24] avoid this assumption but only derive sharp bounds on harm measures, which may yield conservative policies; multiple-experiment approaches [50,51] identify the joint distribution by combining evidence from multiple randomized experiments under weaker assumptions, but they require multiple experimental datasets that are not always available; and structural causal model approaches [12] rely on fully specified causal graphs and structural equations. In contrast, our approach provides point identification under a single observational or experimental dataset by imposing an additive latent-variable model. While this assumption is stronger than those used in bounds-based or multiple-experiment approaches, it yields tractable estimators and enables direct policy learning with explicit harm constraints.

In this article, we introduce the concept of treatment harm quantity (THQ) for continuous outcomes, complementing THR by quantifying the magnitude of counterfactual harm. We establish the identifiability of THR and THQ under an additive latent variable model. The resulting identification is conditional on this model rather than fully nonparametric. Building on these, we further develop a comprehensive counterfactual harm-aware policy learning approach with effectiveness and harm constraints.

## 7. Conclusions

In this work, we address an important yet underexplored aspect of offline policy evaluation and learning: individual-level counterfactual harm. While conventional methods emphasize reward maximization, they may inadvertently produce policies that pose significant risk to individuals. Motivated by the need for trustworthy decision-making that explicitly accounts for counterfactual harm, particularly in high-stakes domains such as healthcare and finance, we analyze the limitations of existing methods, introduce two harm metrics for continuous outcomes, THR and THQ, and establish their identifiability under a novel additive model assumption. We also develop consistent estimators for these metrics and incorporate them into policy learning through constraints on estimated harm. This substantially extends previous methodologies developed for binary outcomes. While the additive model assumption is flexible and avoids the strong conditional independence assumption, it imposes certain restrictions on the data-generating process of the potential outcomes. Further research is needed to explore ways to relax this assumption.

A key limitation of our approach is that Assumption 2 is fundamentally untestable from observed data, because it concerns the joint distribution of two potential outcomes, only one of which is observed for each individual. The plausibility of the assumption therefore must be justified by substantive domain knowledge rather than empirical goodness-of-fit. When the assumption is questionable, the proposed estimators should be interpreted with caution, and sensitivity analyses over the cross-world dependence parameter, as in Section 5.2, can be used to assess the range of plausible harm estimates. Extending the framework to weaker identification assumptions is an important direction for future work.

In addition, we note that under finite-sample settings the realized harm rate and harm quantity may slightly exceed their pre-specified thresholds, even though the proposed method substantially reduces harm relative to unconstrained policies. This gap arises from estimation uncertainty in the nuisance functions and the learned policy. Developing optimization strategies to explicitly remedy this deficiency is an important direction for future work; for example, one could optimize a conservative upper confidence bound on the estimated harm or adopt a distributionally robust constraint that accounts for estimation uncertainty. We leave this as a promising avenue for future research.

A fixed δ is a population-level risk budget: it constrains THR(π) or THQ(π), while the policy π(X) remains individualized. Since counterfactual harm is not directly observable, δ cannot be calibrated on held-out data. To aid in threshold selection, we recommend conducting a sensitivity analysis: for a range of δ, we report the estimated reward–harm trade-off under our identifying assumptions, allowing decision-makers to choose a policy consistent with their risk preferences.

## Figures and Tables

**Figure 1:**
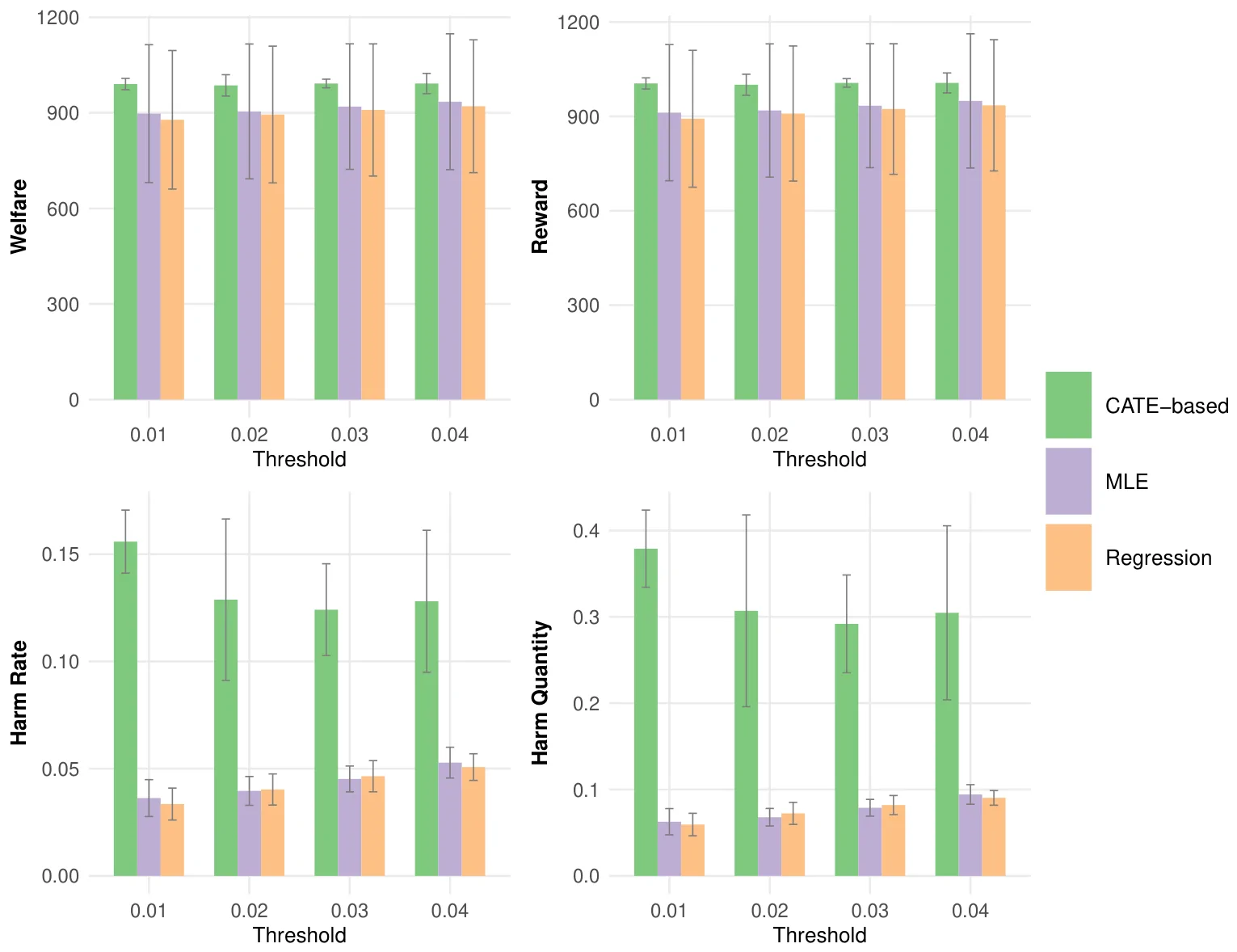
Results of MLE-based and regression-based methods for estimating σ(X,t) on Ihdp.

**Figure 2:**
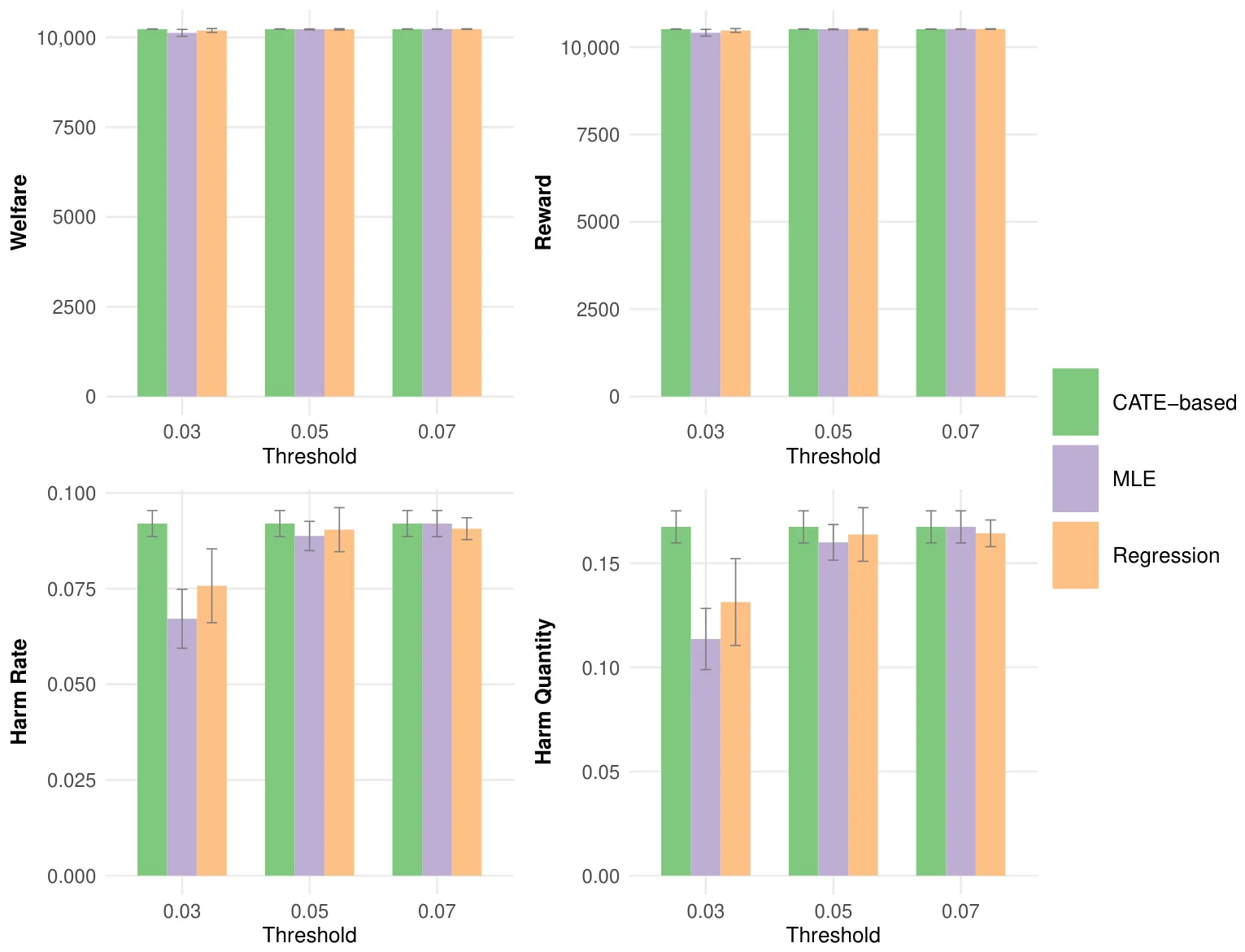
Results of MLE-based and regression-based methods for estimating σ(X,t) on Twins.

**Figure 3:**
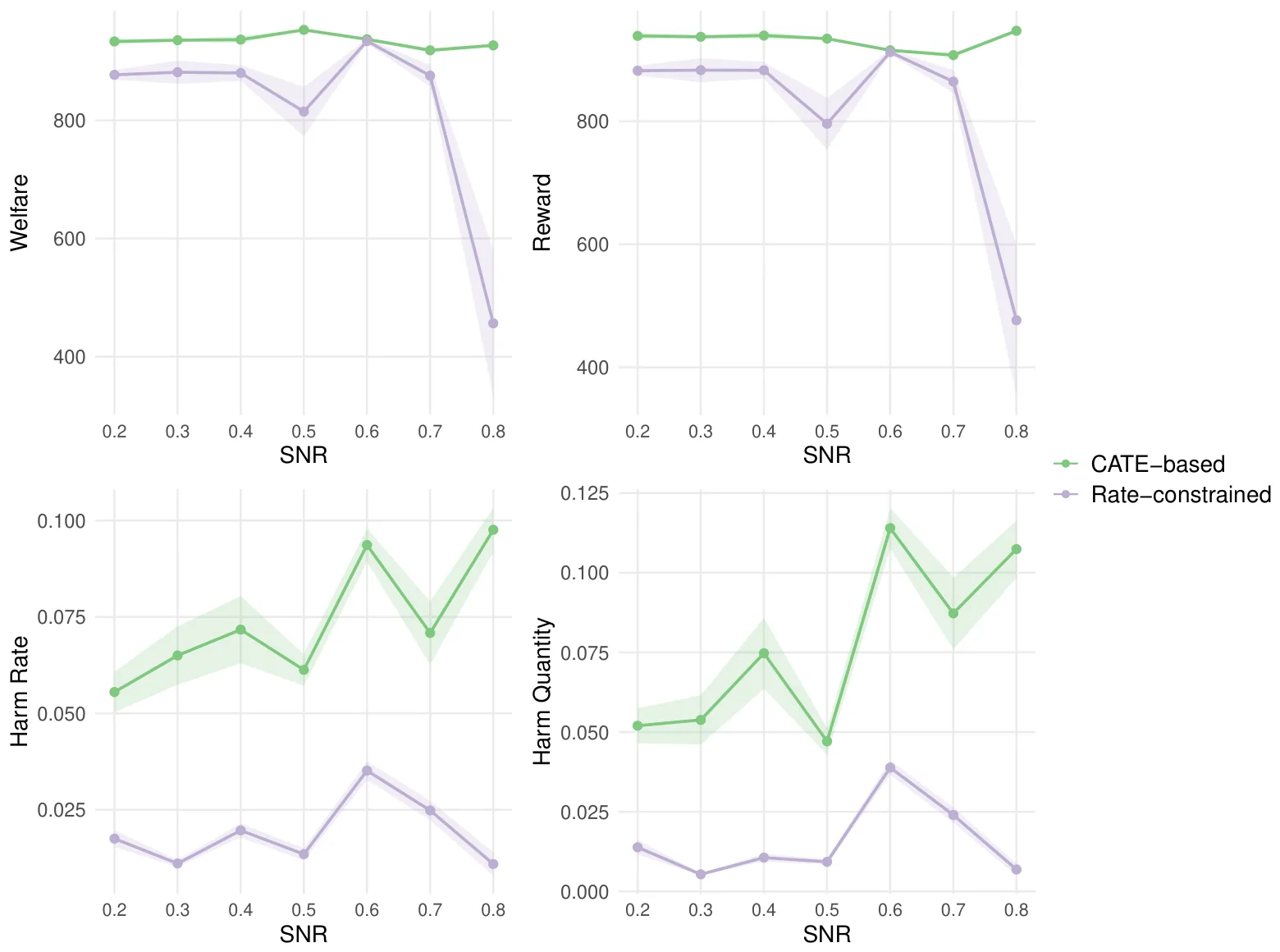
Results under different SNR levels on Ihdp.

**Figure 4:**
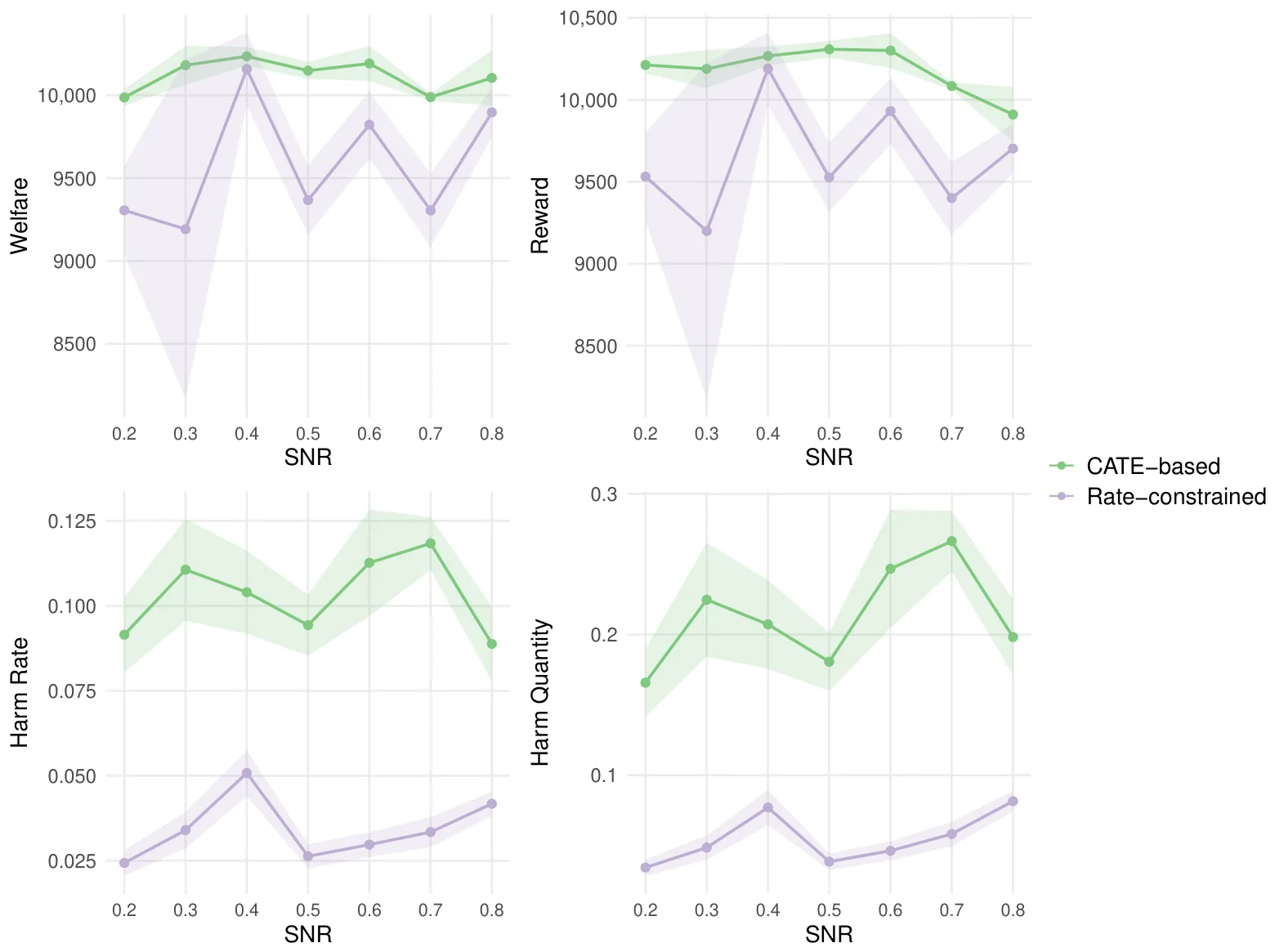
Results under different SNR levels on Twins.

**Table 1:** Performance under different harm rate thresholds.

Ihdp	ΔWelfare	Reward	True Harm Rate	True Harm Quantity
Naive	48.329 ± 0.000	62.428 ± 0.000	0.484 ± 0.000	1.953 ± 0.000
CATE-based	1036.835 ± 2.414	1050.934 ± 2.414	0.138 ± 0.001	0.320 ± 0.004
Rate (δ=0.03)	970.015 ± 52.690	984.114 ± 52.690	0.050 ± 0.002	0.109 ± 0.004
Rate (δ=0.04)	986.277 ± 35.402	1000.376 ± 35.403	0.059 ± 0.002	0.134 ± 0.005
Rate (δ=0.05)	992.690 ± 20.788	1006.790 ± 20.788	0.064 ± 0.002	0.150 ± 0.007
**Twins**	Δ **Welfare**	**Reward**	**True Harm Rate**	**True Harm Quantity**
Naive	−315.562 ± 0.000	−28.272 ± 0.000	0.5083 ± 0.0000	2.3258 ± 0.0000
CATE-based	10,227.300 ± 5.649	10,514.590 ± 5.649	0.0920 ± 0.0034	0.1675 ± 0.0077
Rate (δ=0.02)	9475.085 ± 167.414	9762.376 ± 167.414	0.0458 ± 0.0032	0.0768 ± 0.0052
Rate (δ=0.03)	9704.447 ± 73.441	9991.737 ± 73.441	0.0569 ± 0.0015	0.0986 ± 0.0042
Rate (δ=0.04)	9738.862 ± 178.766	10,026.152 ± 178.766	0.0674 ± 0.0043	0.1225 ± 0.0071

**Table 2:** Sensitivity to non-normal noise on Ihdp and Twins.

Dataset	Setting	Method	ΔWelfare	Reward	True Harm Rate	True Harm Quantity
Ihdp	Uniform noise	Naive	9.222 ± 0.000	26.478 ± 0.000	0.4688 ± 0.0000	1.8866 ± 0.0000
Ihdp	Uniform noise	CATE-based	1201.294 ± 15.766	1218.550 ± 15.766	0.0498 ± 0.0117	0.0604 ± 0.0121
Ihdp	Uniform noise	Rate	1124.490 ± 194.004	1141.747 ± 194.004	0.0340 ± 0.0043	0.0811 ± 0.0100
Ihdp	Exponential noise	Naive	−504.659 ± 0.000	−1022.259 ± 0.000	0.5387 ± 0.0000	2.6280 ± 0.0000
Ihdp	Exponential noise	CATE-based	1199.587 ± 12.118	681.986 ± 12.118	0.0713 ± 0.0153	0.0770 ± 0.0226
Ihdp	Exponential noise	Rate	1158.893 ± 6.219	641.292 ± 6.219	0.0376 ± 0.0048	0.0710 ± 0.0090
Twins	Uniform noise	Naive	−86.452 ± 0.000	54.424 ± 0.000	0.5031 ± 0.0000	2.1981 ± 0.0000
Twins	Uniform noise	CATE-based	10,409.535 ± 28.460	10,550.410 ± 28.460	0.0718 ± 0.0023	0.0980 ± 0.0043
Twins	Uniform noise	Rate	9915.411 ± 43.918	10,056.287 ± 43.918	0.0466 ± 0.0018	0.0607 ± 0.0030
Twins	Exponential noise	Naive	−51.233 ± 0.000	63.357 ± 0.000	0.5193 ± 0.0000	2.1869 ± 0.0000
Twins	Exponential noise	CATE-based	10,516.525 ± 17.697	10,631.114 ± 17.697	0.0866 ± 0.0024	0.0891 ± 0.0044
Twins	Exponential noise	Rate	10,502.463 ± 17.570	10,617.052 ± 17.570	0.0752 ± 0.0003	0.0730 ± 0.0012

**Table 3:** Sensitivity to alternative dependence between potential outcomes.

Dataset	Setting	Method	ΔWelfare	Reward	True Harm Rate	True Harm Quantity
Ihdp	ρ=0.2	Naive	−14.714 ± 0.000	6.255 ± 0.000	0.4836 ± 0.0000	2.0685 ± 0.0000
Ihdp	ρ=0.2	CATE-based	1285.379 ± 2.458	1306.349 ± 2.458	0.0636 ± 0.0014	0.0853 ± 0.0028
Ihdp	ρ=0.2	Rate	1250.840 ± 8.702	1271.810 ± 8.702	0.0388 ± 0.0008	0.0545 ± 0.0014
Ihdp	ρ=0.8	Naive	9.001 ± 0.000	29.970 ± 0.000	0.4717 ± 0.0000	2.0174 ± 0.0000
Ihdp	ρ=0.8	CATE-based	1296.391 ± 1.995	1317.360 ± 1.995	0.0440 ± 0.0009	0.0535 ± 0.0019
Ihdp	ρ=0.8	Rate	1256.807 ± 8.159	1277.776 ± 8.159	0.0354 ± 0.0008	0.0515 ± 0.0022
Twins	ρ=0.2	Naive	−211.797 ± 0.000	46.714 ± 0.000	0.5071 ± 0.0000	2.2826 ± 0.0000
Twins	ρ=0.2	CATE-based	10,334.041 ± 23.663	10,592.552 ± 23.663	0.0912 ± 0.0015	0.1574 ± 0.0033
Twins	ρ=0.2	Rate	6871.119 ± 4742.354	7129.630 ± 4742.354	0.0422 ± 0.0209	0.0813 ± 0.0209
Twins	ρ=0.8	Naive	83.528 ± 0.000	342.039 ± 0.000	0.5005 ± 0.0000	2.0945 ± 0.0000
Twins	ρ=0.8	CATE-based	10,420.716 ± 12.237	10,679.227 ± 12.237	0.0450 ± 0.0011	0.0482 ± 0.0014
Twins	ρ=0.8	Rate	9860.736 ± 74.039	10,119.247 ± 74.039	0.0317 ± 0.0018	0.0385 ± 0.0023

**Table 4:** Sensitivity to nonlinear variance structures.

Dataset	Setting	Method	ΔWelfare	Reward	True Harm Rate	True Harm Quantity
Ihdp	$\sigma_{a}\left(X\right)=\alpha_{a}+\beta_{a}X_{1}^{2}$	Naive	−35.528 ± 0.000	−77.784 ± 0.000	0.5149 ± 0.0000	1.8134 ± 0.0000
Ihdp	$\sigma_{a}\left(X\right)=\alpha_{a}+\beta_{a}X_{1}^{2}$	CATE-based	1066.006 ± 12.441	1023.751 ± 12.441	0.0480 ± 0.0112	0.0520 ± 0.0141
Ihdp	$\sigma_{a}\left(X\right)=\alpha_{a}+\beta_{a}X_{1}^{2}$	Rate	907.137 ± 150.842	864.882 ± 150.842	0.0181 ± 0.0023	0.0172 ± 0.0029
Ihdp	$\sigma_{a}\left(X\right)=\alpha_{a}+\beta_{a}\left|X_{1}\right|+0.2X_{2}^{2}$	Naive	62.434 ± 0.000	139.457 ± 0.000	0.4747 ± 0.0000	1.7591 ± 0.0000
Ihdp	$\sigma_{a}\left(X\right)=\alpha_{a}+\beta_{a}\left|X_{1}\right|+0.2X_{2}^{2}$	CATE-based	1015.054 ± 26.684	1092.077 ± 26.684	0.1429 ± 0.0241	0.2718 ± 0.0580
Ihdp	$\sigma_{a}\left(X\right)=\alpha_{a}+\beta_{a}\left|X_{1}\right|+0.2X_{2}^{2}$	Rate	999.590 ± 164.459	1076.613 ± 164.459	0.0337 ± 0.0031	0.0514 ± 0.0044
Twins	$\sigma_{a}\left(X\right)=\alpha_{a}+\beta_{a}X_{1}^{2}$	Naive	−59.360 ± 0.000	179.095 ± 0.000	0.5035 ± 0.0000	2.2101 ± 0.0000
Twins	$\sigma_{a}\left(X\right)=\alpha_{a}+\beta_{a}X_{1}^{2}$	CATE-based	10,345.780 ± 26.276	10,584.235 ± 26.276	0.0691 ± 0.0019	0.1099 ± 0.0033
Twins	$\sigma_{a}\left(X\right)=\alpha_{a}+\beta_{a}X_{1}^{2}$	Rate	9696.144 ± 81.974	9934.598 ± 81.974	0.0389 ± 0.0016	0.0631 ± 0.0024
Twins	$\sigma_{a}\left(X\right)=\alpha_{a}+\beta_{a}\left|X_{1}\right|+0.2X_{2}^{2}$	Naive	−99.581 ± 0.000	213.985 ± 0.000	0.5075 ± 0.0000	2.2190 ± 0.0000
Twins	$\sigma_{a}\left(X\right)=\alpha_{a}+\beta_{a}\left|X_{1}\right|+0.2X_{2}^{2}$	CATE-based	10,337.655 ± 38.571	10,651.221 ± 38.571	0.0890 ± 0.0013	0.1387 ± 0.0034
Twins	$\sigma_{a}\left(X\right)=\alpha_{a}+\beta_{a}\left|X_{1}\right|+0.2X_{2}^{2}$	Rate	6912.420 ± 4766.553	7225.985 ± 4766.553	0.0368 ± 0.0175	0.0640 ± 0.0120

**Table 5:** Sensitivity to nuisance-model misspecification on Ihdp and Twins.

Dataset	Setting	Method	ΔWelfare	Reward	True Harm Rate	True Harm Quantity
Ihdp	Nonlinear outcome mean	Naive	140.492 ± 0.000	438.658 ± 0.000	0.4568 ± 0.0000	1.9432 ± 0.0000
Ihdp	Nonlinear outcome mean	CATE-based	1360.506 ± 3.651	1658.672 ± 3.651	0.0473 ± 0.0019	0.0646 ± 0.0046
Ihdp	Nonlinear outcome mean	Rate	1321.423 ± 8.377	1619.589 ± 8.377	0.0355 ± 0.0011	0.0532 ± 0.0016
Twins	Nonlinear outcome mean	Naive	1203.002 ± 0.000	2794.799 ± 0.000	0.4849 ± 0.0000	2.0748 ± 0.0000
Twins	Nonlinear outcome mean	CATE-based	11,057.210 ± 23.230	12,649.008 ± 23.230	0.0822 ± 0.0025	0.1209 ± 0.0048
Twins	Nonlinear outcome mean	Rate	11,024.409 ± 18.831	12,616.207 ± 18.831	0.0712 ± 0.0014	0.1030 ± 0.0026

## Data Availability

The Ihdp and Twins datasets used in this study are publicly available from the archived CEVAE GitHub repository at https://github.com/AMLab-Amsterdam/CEVAE/tree/master/datasets/IHDP (accessed on 20 July 2026) and https://github.com/AMLab-Amsterdam/CEVAE/tree/master/datasets/TWINS (accessed on 20 July 2026), respectively. The code generated in this study is available from the corresponding author upon reasonable request.

## Appendix A

**Proof of Theorem 1.** To identify THR(x) and THQ(x), we first identify the conditional distribution $P(Y^{1}-Y^{0}|X=x)$. Recall that we assume the potential outcomes take the form of $Y^{t}=\mu \left(x,t\right)+\sigma \left(x,t\right)U,\;\sigma_{t}>0,\;t\in \left\{0,1\right\}$, with U∣X∼N(0,1), in Assumption 2, which implies that

$$\begin{array}{c} Y^{1}-Y^{0}|X=x∼N\left(\mu \left(x,1\right)-\mu \left(x,0\right),{\left(\sigma (x,1)-\sigma (x,0)\right)}^{2}\right). \end{array}$$

To identify $P(Y^{1}-Y^{0}|X=x)$, it suffices to identify μ(x,1)−μ(x,0) and ${\left(\sigma (x,1)-\sigma (x,0)\right)}^{2}$. Note that the conditional mean of $Y^{t}$ given X=x is

$$\begin{array}{c} E\left(Y^{t}|X=x\right)=E\left(\mu \left(x,t\right)+\sigma \left(x,t\right)U|X=x\right)=\mu \left(x,t\right). \end{array}$$

The conditional mean $E(Y^{t}|X=x)$ can be identified as E(Y∣X=x,T=t) under Assumption 1, which leads to the identifiability of μ(x,t) for t=0,1. We also note that the conditional variance of $Y^{t}$ given X=x is

$$\begin{array}{cc} \; & \mathrm{Var}(Y^{t}|X=x)\\ = & E\left({\left(\mu \left(x,t\right)+\sigma \left(x,t\right)U\right)}^{2}|X=x\right)-{\left(E\left(Y^{t}|X=x\right)\right)}^{2}\\ = & E\left(\mu^{2}\left(x,t\right)+2\mu \left(x,t\right)\sigma \left(x,t\right)U+\sigma^{2}\left(x,t\right)U^{2}|X=x\right)-\mu^{2}\left(x,t\right)\\ = & \sigma^{2}\left(x,t\right). \end{array}$$

Similarly, $\mathrm{Var}(Y^{t}|X=x)$ can be identified under Assumption 1. This leads to the identifiability of σ(x,t) for t=0,1, and thus ${\left(\sigma (x,1)-\sigma (x,0)\right)}^{2}$ is identifiable. Then, we prove (i). When σ(x,1)≠σ(x,0), since $Y^{1}-Y^{0}|X=x∼N\left(\mu \left(x,1\right)-\mu \left(x,0\right),{\left(\sigma (x,1)-\sigma (x,0)\right)}^{2}\right)$, we have

$$\begin{array}{cc} \mathrm{THR}(x)= & P\left(Y^{1}-Y^{0}<0|X=x\right)\\ = & \int_{-\infty }^{0}\frac{1}{\sqrt{2\pi }\left|\sigma \left(x,1\right)-\sigma \left(x,0\right)\right|}\exp\left\{\frac{u-(\mu (x,1)-\mu (x,0))}{\left|\sigma (x,1)-\sigma (x,0)\right|}\right\}du\\ = & \int_{-\infty }^{-(\mu (x,1)-\mu (x,0))/|\sigma (x,1)-\sigma (x,0)|}\frac{1}{\sqrt{2\pi }}\exp\left(-\frac{1}{2}t^{2}\right)dt\\ = & \Phi \left(\frac{\mu (x,0)-\mu (x,1)}{\left|\sigma (x,1)-\sigma (x,0)\right|}\right), \end{array}$$

where Φ(·) denotes the standard normal cumulative distribution function. Let Z∼N(0,1), and denote $a=\mu \left(x,1\right)-\mu \left(x,0\right),\;b=\left|\sigma (x,1)-\sigma (x,0)\right|>0$. Then,

$$\begin{array}{cc} \; & \mathrm{THQ}\left(x\right)=E\left[\left(Y^{0}-Y^{1}\right)I_{\left\{Y^{1}-Y^{0}<0\right\}}|X=x\right]\\ = & -E[\left(Y^{1}-Y^{0}\right)I_{\left\{Y^{1}-Y^{0}<0\right\}}|X=x]\\ = & -\int_{-\infty }^{0}rf_{Y^{1}-Y^{0}|X=x}\left(r\right)\;dr\\ = & -\int_{-\infty }^{0}r\frac{1}{\sqrt{2\pi }b}\exp\left\{-\frac{1}{2b^{2}}{\left(r-a\right)}^{2}\right\}\;dr\\ = & -\int_{-\infty }^{-\frac{a}{b}}\left(a+bz\right)\frac{1}{\sqrt{2\pi }}\exp\left\{-\frac{1}{2}z^{2}\right\}\;dr\\ = & -a\Phi \left(-\frac{a}{b}\right)+\frac{b}{\sqrt{2\pi }}\exp\left\{-\frac{a^{2}}{2b^{2}}\right\}\\ = & \left(\mu (x,0)-\mu (x,1)\right)\Phi \left(\frac{\mu (x,0)-\mu (x,1)}{\left|\sigma (x,1)-\sigma (x,0)\right|}\right)\\ & +\frac{\left|\sigma (x,1)-\sigma (x,0)\right|}{\sqrt{2\pi }}\exp\left\{-\frac{{\left(\mu \left(x,1\right)-\mu \left(x,0\right)\right)}^{2}}{2{\left(\sigma (x,1)-\sigma (x,0)\right)}^{2}}\right\}. \end{array}$$

This completes the proof of (i). Next, we prove (ii). When σ(x,1)=σ(x,0), $Y^{1}-Y^{0}|X=x$ reduces to μ(x,1)−μ(x,0), according to the preceding proof. This implies $P\left(Y^{1}-Y^{0}|X=x\right)$ can be identified under Assumption 1. Then we consider the identification of THR(x) and THQ(x).

$$\begin{array}{cc} \mathrm{THR}(x)= & P\left(Y^{1}-Y^{0}<0|X=x\right)\\ = & \left\{\begin{array}{cc} 0, & \;\mathrm{if}\;\;\mu (x,1)-\mu (x,0)⩾0\\ 1, & \;\mathrm{if}\;\;\mu (x,1)-\mu (x,0)<0, \end{array}\right. \end{array}$$

$$\begin{array}{cc} \; & \mathrm{THQ}\left(x\right)=E\left[\left(Y^{0}-Y^{1}\right)I_{\left\{Y^{1}-Y^{0}<0\right\}}|X=x\right]\\ = & -E[\left(Y^{1}-Y^{0}\right)I_{\left\{Y^{1}-Y^{0}<0\right\}}|X=x]\\ = & \left\{\begin{array}{cc} 0, & \;\mathrm{if}\;\;\mu (x,1)-\mu (x,0)⩾0\\ \mu (x,0)-\mu (x,1), & \;\mathrm{if}\;\;\mu (x,1)-\mu (x,0)<0. \end{array}\right. \end{array}$$

This completes the proof of (ii). □

## Appendix B. Details for Policy Learning with Harm Constraints

**Proof.** **Maximum likelihood estimator for σ(x,t)**. Given $\hat{\mu }\left(x,t\right)$ for t=0,1, if we parameterize σ(x,t) as $\sigma_{t}\left(x,\beta_{t}\right)$, then the likelihood of the observed data is given by

$$\begin{array}{cc} L(\beta_{0},\beta_{1})= & \prod_{i=1}^{n}{\left[\frac{1}{\sqrt{2\pi }\sigma^{2}\left(X_{i},1,\beta_{1}\right)}\exp\left(-\frac{1}{2\sigma^{2}\left(X_{i},1,\beta_{1}\right)}{\left(Y_{i}-\hat{\mu }\left(X_{i},1\right)\right)}^{2}\right)\right]}^{T_{i}}\\ & \times \prod_{i=1}^{n}{\left[\frac{1}{\sqrt{2\pi }\sigma^{2}\left(X_{i},0,\beta_{0}\right)}\exp\left(-\frac{1}{2\sigma^{2}\left(X_{i},0,\beta_{0}\right)}{\left(Y_{i}-\hat{\mu }\left(X_{i},0\right)\right)}^{2}\right)\right]}^{1-T_{i}}. \end{array}$$

Therefore, the log-likelihood is

$$\begin{array}{cc} l(\beta_{0},\beta_{1})= & \log L(\beta_{0},\beta_{1})\\ = & -\frac{n}{2}\log\left(2\pi \right)-\sum_{i=1}^{n}T_{i}\left\{\log\sigma^{2}\left(X_{i},1,\beta_{1}\right)+\frac{1}{2\sigma^{2}\left(X_{i},1,\beta_{1}\right)}{\left(Y_{i}-\hat{\mu }\left(X_{i},1\right)\right)}^{2}\right\}\\ & -\sum_{i=1}^{n}\left(1-T_{i}\right)\left\{\log\sigma^{2}\left(X_{i},0,\beta_{0}\right)+\frac{1}{2\sigma^{2}\left(X_{i},0,\beta_{0}\right)}{\left(Y_{i}-\hat{\mu }\left(X_{i},0\right)\right)}^{2}\right\}. \end{array}$$

**Regression-based method for estimating σ(x,t)**. Under Assumption 2, we have

$$\begin{array}{cc} \sigma^{2}\left(x,t\right)= & \mathrm{Var}[Y^{t}|X=x]\\ = & E[{\left\{Y^{t}-E\left(Y^{t}|X\right)\right\}}^{2}|X=x]\\ = & E[{\left\{Y^{t}-\mu \left(X,t\right)\right\}}^{2}|X=x]. \end{array}$$

In addition, under Assumption 1,

$$E\left[{\left\{Y^{t}-\mu \left(X,t\right)\right\}}^{2}|X=x\right]=E\left[{\left\{Y^{t}-\mu \left(X,t\right)\right\}}^{2}|X=x,T=t\right].$$

Thus, given $\hat{\mu }\left(x,t\right)$, we can estimate $\sigma^{2}\left(x,t\right)$ by regressing ${\left(Y-\hat{\mu }\left(X,t\right)\right)}^{2}$ on *X* using the data with T=t. **Discussion of the optimization problem**. Below, we provide a detailed discussion on how to solve the following optimization problem

$$\begin{array}{c} \left\{\begin{array}{c} \max_{\pi \in \Pi }\hat{R}\left(\pi \right)\\ s.t.\;\hat{\mathrm{THR}}\left(\pi \right)⩽\delta , \end{array}\right. \end{array}$$

where

$$\begin{array}{cc} \hat{R}\left(\pi \right)= & n^{-1}\sum_{i=1}^{n}\left[\varphi_{\pi }\left(Z_{i};\hat{e},\hat{\mu }\left(0\right),\hat{\mu }\left(1\right)\right)\right],\\ \hat{\mathrm{THR}}\left(\pi \right)= & n^{-1}\sum_{i=1}^{n}\hat{\mathrm{THR}}\left(X_{i}\right)\cdot \pi \left(X_{i}\right), \end{array}$$

with

$$\begin{array}{cc} \varphi_{\pi }\left(Z_{i};\hat{e},\hat{\mu }\left(0\right),\hat{\mu }\left(1\right)\right)= & \left(\frac{T_{i}\left(Y_{i}-\hat{\mu }\left(X_{i},1\right)\right)}{\hat{e}\left(X_{i}\right)}+\hat{\mu }\left(X_{i},1\right)\right)\pi \left(X_{i}\right)\\ \; & +\left(\frac{\left(1-T_{i}\right)\left(Y_{i}-\hat{\mu }\left(X_{i},0\right)\right)}{1-\hat{e}\left(X_{i}\right)}+\hat{\mu }\left(X_{i},0\right)\right)\left(1-\pi \left(X_{i}\right)\right). \end{array}$$

Assuming the model π(x) is parameterized as π(x,θ), we propose to solve for the parameter θ using the Lagrange multiplier method. Specifically, we first note that

$$\begin{array}{cc} \hat{\pi }= & \left\{\begin{array}{c} \arg\max_{\pi \in \Pi }\;n^{-1}\sum_{i=1}^{n}\varphi_{\pi }\left(Z_{i};\hat{e},\hat{\mu }\left(0\right),\hat{\mu }\left(1\right)\right)\\ s.t.\;n^{-1}\sum_{i=1}^{n}\hat{\mathrm{THR}}\left(X_{i}\right)\cdot \pi \left(X_{i}\right)⩽\delta \end{array}\right.\\ = & \left\{\begin{array}{c} \arg\max_{\pi \in \Pi }\;n^{-1}\sum_{i=1}^{n}\hat{\tau }\left(X_{i}\right)\pi \left(X_{i}\right)\\ s.t.\;n^{-1}\sum_{i=1}^{n}\hat{\mathrm{THR}}\left(X_{i}\right)\cdot \pi \left(X_{i}\right)⩽\delta , \end{array}\right. \end{array}$$

where

$$\begin{array}{cc} \hat{\tau }\left(X_{i}\right)= & \left(\frac{T_{i}\left(Y_{i}-\hat{\mu }\left(X_{i},1\right)\right)}{\hat{e}\left(X_{i}\right)}+\hat{\mu }\left(X_{i},1\right)\right)-\left(\frac{\left(1-T_{i}\right)\left(Y_{i}-\hat{\mu }\left(X_{i},0\right)\right)}{1-\hat{e}\left(X_{i}\right)}+\hat{\mu }\left(X_{i},0\right)\right). \end{array}$$

On the one hand, if $\delta ⩾n^{-1}\sum_{i=1}^{n}\hat{\mathrm{THR}}\left(X_{i}\right)I\left(\hat{\tau }\left(X_{i}\right)>0\right)$, then the constraint $n^{-1}\sum_{i=1}^{n}\hat{\mathrm{THR}}\left(X_{i}\right)\pi \left(X_{i}\right)⩽\lambda$ becomes inactive, and the constrained optimization problem turns into an unconstrained optimization problem. That is, we could solve π(x,θ) by maximizing

$$n^{-1}\sum_{i=1}^{n}\hat{\tau }\left(X_{i}\right)\pi \left(X_{i},\theta \right).$$

On the other hand, if $\delta <n^{-1}\sum_{i=1}^{n}\hat{\mathrm{THR}}\left(X_{i}\right)I\left(\hat{\tau }\left(X_{i}\right)>0\right)$, the optimal solution is obtained at the boundary of the constraint. By introducing the Lagrange multiplier β that satisfies

$$n^{-1}\sum_{i=1}^{n}\hat{\mathrm{THR}}\left(X_{i}\right)\cdot I\left(\hat{\tau }\left(X_{i}\right)-\beta \hat{\mathrm{THR}}\left(X_{i}\right)>0\right)=\delta ,$$

and we could learn π(x,θ) by maximizing

$$n^{-1}\sum_{i=1}^{n}\pi \left(X_{i},\theta \right)\left(\hat{\tau }\left(X_{i}\right)-\beta \hat{\mathrm{THR}}\left(X_{i}\right)\right).$$

□

## Appendix C

**Proof of Theorem 2.** For clarity, we summarize the conditions in the theorem as follows: C1: Π is a P-G-C class. C2: $\hat{\mu }\left(x,t\right)$, $\hat{\sigma }\left(x,t\right)$ and $\hat{e}\left(x\right)$ are uniformly consistent estimators of μ(x,t), σ(x,t), and e(x) for t=0,1. C3: ∀π∈Π,a∈(0,1),aπ∈Π. Under these conditions, we have

$$\begin{array}{cc} & {\hat{\pi }}^{*}=\arg\max_{\pi \in \Pi }\hat{R}\left(\pi \right)=\arg\max_{\pi \in \Pi }\frac{1}{n}\sum_{i=1}^{n}\varphi_{\pi }\left(T_{i},X_{i},Y_{i};\hat{e},\hat{\mu }\left(0\right),\hat{\mu }\left(1\right)\right)\\ & \;\;s.t.\;\;\frac{1}{n}\sum_{i=1}^{n}\psi_{\pi }\left(T_{i},X_{i},Y_{i};\hat{e},\hat{\mu }\left(0\right),\hat{\mu }\left(1\right)\right)⩽\lambda ,\\ & \pi^{*}=\arg\max_{\pi \in \Pi }R\left(\pi \right)=\arg\max_{\pi \in \Pi }E\psi_{\pi }\left(T,X,Y;e,\mu (0),\mu (1)\right)\\ & \;\;s.t.\;\;\;E\left[\psi_{\pi }\left(T_{i},X_{i},Y_{i};e,\mu \left(0\right),\mu \left(1\right)\right)\right]⩽\lambda , \end{array}$$

where

$$\begin{array}{cc} \; & \varphi_{\pi }\left(T,X,Y;e,\mu (0),\mu (1)\right)\\ = & \left(\frac{T\left(Y-\mu (X,1)\right)}{e(X)}+\mu \left(X,1\right)\right)\pi \left(X\right)+\left(\frac{\left(1-T\right)\left(Y-\mu (X,0)\right)}{1-e(X)}+\mu \left(X,0\right)\right)\left(1-\pi \left(X\right)\right), \end{array}$$

$$\begin{array}{cc} \; & \psi_{\pi }\left(T,X,Y;e,\mu (0),\mu (1)\right)\\ = & \left(\frac{\left(1-T\right)\left(Y-\mu (X,0)\right)}{1-e(X)}+\mu \left(X,0\right)\right)\pi \left(X\right)-\left(\frac{T\left(Y-\mu (X,1)\right)}{e(X)}+\mu \left(X,1\right)\right)\mu \left(X,0\right)\pi \left(X\right). \end{array}$$

Note that $\frac{T\left(Y-\mu (X,1)\right)}{e(X)}+\mu \left(X,1\right)$, $\frac{\left(1-T\right)\left(Y-\mu (X,0)\right)}{1-e(X)}+\mu \left(X,0\right)$, $\left(\frac{T\left(Y-\mu (X,1)\right)}{e(X)}+\mu \left(X,1\right)\right)$ μ(X,0) are bounded random variables, and both $\left\{\varphi_{\pi }:\pi \in \Pi \right\}$ and $\left\{\psi_{\pi }:\pi \in \Pi \right\}$ are P-G-C classes by condition C2 and Theorem 9.26 in Kosorok [53]. First, we prove (a). Let

$$\begin{array}{cc} \Pi_{n,\lambda } & =\left\{\pi \in \Pi |P_{n}\psi_{\pi }\left(T,X,Y;\hat{e},\hat{\mu }\left(0\right),\hat{\mu }\left(1\right)\right)⩽\lambda \right\},\\ \Pi_{\lambda } & =\left\{\pi \in \Pi |E\psi_{\pi }\left(T,X,Y;e,\mu (0),\mu (1)\right)⩽\lambda \right\}, \end{array}$$

where $P_{n}$ means sample average, then we have

$$\hat{R}\left({\hat{\pi }}^{*}\right)-R\left(\pi^{*}\right)=-\left(D_{1n}+D_{2n}+D_{3n}\right),$$

where

$$\begin{array}{cc} D_{1n} & =\max_{\pi \in \Pi_{\lambda }}E\varphi_{\pi }\left(T,X,Y;e,\mu (0),\mu (1)\right)-\max_{\pi \in \Pi_{\lambda }}P_{n}\varphi_{\pi }\left(T,X,Y;e,\mu (0),\mu (1)\right),\\ D_{2n} & =\max_{\pi \in \Pi_{\lambda }}P_{n}\varphi_{\pi }\left(T,X,Y;e,\mu (0),\mu (1)\right)-\max_{\pi \in \Pi_{\lambda }}P_{n}\varphi_{\pi }\left(T,X,Y;\hat{e},\hat{\mu }\left(0\right),\hat{\mu }\left(1\right)\right),\\ D_{3n} & =\max_{\pi \in \Pi_{\lambda }}P_{n}\varphi_{\pi }\left(T,X,Y;\hat{e},\hat{\mu }\left(0\right),\hat{\mu }\left(1\right)\right)-\max_{\pi \in \Pi_{n,\lambda }}P_{n}\varphi_{\pi }\left(T,X,Y;\hat{e},\hat{\mu }\left(0\right),\hat{\mu }\left(1\right)\right). \end{array}$$

We discuss $D_{in},\;i=1,2,3$ one by one:

$$\begin{array}{cc} D_{1n} & =\max_{\pi \in \Pi_{\lambda }}E\varphi_{\pi }\left(T,X,Y;e,\mu (0),\mu (1)\right)-\max_{\pi \in \Pi_{\lambda }}P_{n}\varphi_{\pi }\left(T,X,Y;e,\mu (0),\mu (1)\right)\\ & ⩽\max_{\pi \in \Pi_{\lambda }}\left|E\varphi_{\pi }\left(T,X,Y;e,\mu (0),\mu (1)\right)-P_{n}\varphi_{\pi }\left(T,X,Y;e,\mu (0),\mu (1)\right)\right|\\ & =o_{P}\left(1\right), \end{array}$$

where the last equality follows since $\left\{\varphi_{\pi }:\pi \in \Pi \right\}$ is a P-G-C class.

$$\begin{array}{cc} D_{2n} & =\max_{\pi \in \Pi_{\lambda }}P_{n}\varphi_{\pi }\left(T,X,Y;e,\mu (0),\mu (1)\right)-\max_{\pi \in \Pi_{\lambda }}P_{n}\varphi_{\pi }\left(T,X,Y;\hat{e},\hat{\mu }\left(0\right),\hat{\mu }\left(1\right)\right)\\ & ⩽\max_{\pi \in \Pi_{\lambda }}\left|P_{n}\left[\varphi_{\pi }\left(T,X,Y;e,\mu (0),\mu (1)\right)-\varphi_{\pi }\left(T,X,Y;\hat{e},\hat{\mu }\left(0\right),\hat{\mu }\left(1\right)\right)\right]\right|\\ & =o_{P}\left(1\right), \end{array}$$

where the last equality follows from Condition C2. ∀ε>0,∃N∈N, such that ∀n>N

$$\begin{array}{cc} D_{3n} & =\max_{\pi \in \Pi_{\lambda }}P_{n}\varphi_{\pi }\left(T,X,Y;\hat{e},\hat{\mu }\left(0\right),\hat{\mu }\left(1\right)\right)-\max_{\pi \in \Pi_{n,\lambda }}P_{n}\varphi_{\pi }\left(T,X,Y;\hat{e},\hat{\mu }\left(0\right),\hat{\mu }\left(1\right)\right)\\ & ⩽\max_{\pi \in \Pi_{\lambda }}P_{n}\varphi_{\pi }\left(T,X,Y;\hat{e},\hat{\mu }\left(0\right),\hat{\mu }\left(1\right)\right)-\max_{\pi \in \frac{\lambda }{\lambda +\varepsilon }\Pi_{\lambda }}P_{n}\varphi_{\pi }\left(T,X,Y;\hat{e},\hat{\mu }\left(0\right),\hat{\mu }\left(1\right)\right)\\ & ⩽\frac{\varepsilon }{\lambda +\varepsilon }L. \end{array}$$

The first inequality follows from the following explanation. For ∀π∈Π,

$$\begin{array}{cc} & E\psi_{\pi }\left(T,X,Y;e,\mu (0),\mu (1)\right)-P_{n}\psi_{\pi }\left(T,X,Y;\hat{e},\hat{\mu }\left(0\right),\hat{\mu }\left(1\right)\right)\\ = & E\psi_{\pi }\left(T,X,Y;e,\mu (0),\mu (1)\right)-P_{n}\psi_{\pi }\left(T,X,Y;e,\mu (0),\mu (1)\right)\\ & +P_{n}\psi_{\pi }\left(T,X,Y;e,\mu (0),\mu (1)\right)-P_{n}\psi_{\pi }\left(T,X,Y;\hat{e},\hat{\mu }\left(0\right),\hat{\mu }\left(1\right)\right), \end{array}$$

converges to zero by conditions C1 and C2. Thus, ∀ε>0,∃N∈N, such that ∀n>N,

$$\begin{array}{c} \left|E\psi_{\pi }\left(T,X,Y;e,\mu (0),\mu (1)\right)-P_{n}\psi_{\pi }\left(T,X,Y;\hat{e},\hat{\mu }\left(0\right),\hat{\mu }\left(1\right)\right)\right|<\varepsilon , \end{array}$$

which implies that $\forall \pi \in \Pi_{\lambda }$, i.e., $E\psi_{\pi }\left(T,X,Y;e,\mu (0),\mu (1)\right)⩽\lambda$, then we have

$$P_{n}\psi_{\pi }\left(T,X,Y;\hat{e},\hat{\mu }\left(0\right),\hat{\mu }\left(1\right)\right)<\lambda +\varepsilon ,$$

which means $\frac{\lambda }{\lambda +\varepsilon }\pi \in \Pi_{n,\lambda },\;i.e.,\;\frac{\lambda }{\lambda +\epsilon }\pi \subseteq \Pi_{n,\lambda }$. The second inequality follows from the following explanation. For any $\pi_{1}$ and $\pi_{2}$,

$$\begin{array}{c} \left|\varphi_{\pi_{1}}\left(T,X,Y;\hat{e},\hat{\mu }\left(0\right),\hat{\mu }\left(1\right)\right)-\varphi_{\pi_{2}}\left(T,X,Y;\hat{e},\hat{\mu }\left(0\right),\hat{\mu }\left(1\right)\right)\right|⩽L\underset{x\in X}{\sup}\left|\pi_{1}\left(x\right)-\pi_{2}\left(x\right)\right|, \end{array}$$

where *L* is a positive constant, since $\varphi_{\pi }$ is uniformly bounded with sufficiently large samples by condition C2. Likewise, for the same ε, $\exists N^{\prime }\in N$, such that $\forall n>n^{\prime }$,

$$\begin{array}{cc} D_{3n} & =\max_{\pi \in \Pi_{\lambda }}P_{n}\varphi_{\pi }\left(T,X,Y;\hat{e},\hat{\mu }\left(0\right),\hat{\mu }\left(1\right)\right)-\max_{\pi \in \Pi_{n,\lambda }}P_{n}\varphi_{\pi }\left(T,X,Y;\hat{e},\hat{\mu }\left(0\right),\hat{\mu }\left(1\right)\right)\\ & ⩾-\frac{\varepsilon }{\lambda +\varepsilon }L. \end{array}$$

The above implies that $D_{3n}=o_{P}\left(1\right)$. This completes the proof of (a). Next, we prove (b). We have

$$R\left({\hat{\pi }}^{*}\right)-R\left(\pi^{*}\right)=-\left(H_{1n}+H_{2n}+H_{3n}\right),$$

where

$$\begin{array}{cc} H_{1n} & =\max_{\pi \in \Pi_{\lambda }}E\varphi_{\pi }\left(T,X,Y;e,\mu (0),\mu (1)\right)-\max_{\pi \in \Pi_{\lambda }}P_{n}\varphi_{\pi }\left(T,X,Y;\hat{e},\hat{\mu }\left(0\right),\hat{\mu }\left(1\right)\right),\\ H_{2n} & =\max_{\pi \in \Pi_{\lambda }}P_{n}\varphi_{\pi }\left(T,X,Y;\hat{e},\hat{\mu }\left(0\right),\hat{\mu }\left(1\right)\right)-\max_{\pi \in \Pi_{n,\lambda }}P_{n}\varphi_{\pi }\left(T,X,Y;\hat{e},\hat{\mu }\left(0\right),\hat{\mu }\left(1\right)\right),\\ H_{3n} & =\max_{\pi \in \Pi_{n,\lambda }}P_{n}\varphi_{\pi }\left(T,X,Y;\hat{e},\hat{\mu }\left(0\right),\hat{\mu }\left(1\right)\right)-\max_{\pi \in \Pi_{n,\lambda }}E\varphi_{\pi }\left(T,X,Y;e,\mu (0),\mu (1)\right). \end{array}$$

We discuss $H_{in},\;i=1,2,3$ one by one: $H_{1n}=o_{P}\left(1\right)$ holds from condition C2. $H_{2n}=D_{3n}=o_{P}\left(1\right)$ follows from the proof above.

$$\begin{array}{cc} H_{3n} & =\max_{\pi \in \Pi_{n,\lambda }}P_{n}\varphi_{\pi }\left(T,X,Y;\hat{e},\hat{\mu }\left(0\right),\hat{\mu }\left(1\right)\right)-\max_{\pi \in \Pi_{n,\lambda }}E\varphi_{\pi }\left(T,X,Y;e,\mu (0),\mu (1)\right)\\ & =P_{n}\varphi_{\hat{\pi }}\left(T,X,Y;\hat{e},\hat{\mu }\left(0\right),\hat{\mu }\left(1\right)\right)-E\varphi_{\hat{\pi }}\left(T,X,Y;e,\mu (0),\mu (1)\right)\\ & =P_{n}\varphi_{\hat{\pi }}\left(T,X,Y;\hat{e},\hat{\mu }\left(0\right),\hat{\mu }\left(1\right)\right)-P_{n}\varphi_{\hat{\pi }}\left(T,X,Y;e,\mu (0),\mu (1)\right)\\ & \;+P_{n}\varphi_{\hat{\pi }}\left(T,X,Y;e,\mu (0),\mu (1)\right)-E\varphi_{\hat{\pi }}\left(T,X,Y;e,\mu (0),\mu (1)\right)\\ & =o_{P}\left(1\right), \end{array}$$

where the last equality follows from

$$\begin{array}{c} P_{n}\varphi_{\hat{\pi }}\left(T,X,Y;\hat{e},\hat{\mu }\left(0\right),\hat{\mu }\left(1\right)\right)-P_{n}\varphi_{\hat{\pi }}\left(T,X,Y;e,\mu (0),\mu (1)\right)=o_{P}\left(1\right), \end{array}$$

by condition C2 and

$$\begin{array}{c} P_{n}\varphi_{\hat{\pi }}\left(T,X,Y;e,\mu (0),\mu (1)\right)-E\left[\varphi_{\hat{\pi }}\left(T,X,Y;e,\mu (0),\mu (1)\right)\right]=o_{P}\left(1\right), \end{array}$$

since $\left\{\varphi_{\pi }:\pi \in \Pi \right\}$ is a P-G-C class. This completes the proof of (b). □

## Appendix D. The General Case with a Cost of Activating Treatment *c*(*x*)

We discuss a more general scenario where activating the treatment incurs a cost c(x)⩾0 in the subpopulation X=x.

**Definition** **A1** (${\mathrm{THR}}_{c(x)}$ and ${\mathrm{THQ}}_{c(x)}$)**.** *The ${\mathrm{THR}}_{c(x)}$ in the subpopulation X=x is defined as*

$${\mathrm{THR}}_{c(x)}\left(x\right)=P\left(Y^{1}-Y^{0}<c\left(x\right)|X=x\right),$$

*and the ${\mathrm{THQ}}_{c(x)}$ in the subpopulation X=x is defined as*

$${\mathrm{THQ}}_{c(x)}\left(x\right)=E\left[\left(Y^{0}-Y^{1}+c\left(x\right)\right)I\left(Y^{1}-Y^{0}<c\left(x\right)\right)|X=x\right].$$

Similarly, the identification of $THR_{c(x)}\left(x\right)$ and $THQ_{c(x)}\left(x\right)$ can be established.

**Theorem** **A1** (Identification of $THR_{c(x)}\left(x\right)$ and $THQ_{c(x)}\left(x\right)$)**.** *Under Assumptions 1 and 2, ${\mathrm{THR}}_{c(x)}\left(x\right)$ and ${\mathrm{THQ}}_{c(x)}\left(x\right)$ are identifiable:*
*(i) when σ(x,1)≠σ(x,0), the identification formulas are:*

$$\begin{array}{cc} {\mathrm{THR}}_{c(x)}\left(x\right)= & \Phi \left(\frac{c(x)-\{\mu (x,1)-\mu (x,0)\}}{\left|\sigma (x,1)-\sigma (x,0)\right|}\right),\\ THQ_{c(x)}\left(x\right)= & \left(c(x)+\mu (x,0)-\mu (x,1)\right)\Phi \left(\frac{c(x)+\mu (x,0)-\mu (x,1)}{\left|\sigma (x,1)-\sigma (x,0)\right|}\right)\\ & +\frac{\left|\sigma (x,1)-\sigma (x,0)\right|}{\sqrt{2\pi }}\exp\left\{-\frac{{\left\{c(x)+\mu (x,0)-\mu (x,1)\right\}}^{2}}{2{\left\{\sigma (x,1)-\sigma (x,0)\right\}}^{2}}\right\}. \end{array}$$

*(ii) when σ(x,1)=σ(x,0), the identification formulas are:*

$$\begin{array}{cc} \; & {\mathrm{THR}}_{c(x)}\left(x\right)=I\left(\mu \left(x,1\right)-\mu \left(x,0\right)<c\left(x\right)\right),\\ \; & {\mathrm{THQ}}_{c(x)}\left(x\right)=\left(c\left(x\right)+\mu \left(x,0\right)-\mu \left(x,1\right)\right)I\left(\mu \left(x,1\right)-\mu \left(x,0\right)<c\left(x\right)\right). \end{array}$$

Theorem A1 establishes the identifiability of ${\mathrm{THR}}_{c(x)}\left(x\right)$ and ${\mathrm{THQ}}_{c(x)}\left(x\right)$, and Theorem A1(ii) is a degenerate case of Theorem A1(i). Similarly, Theorem A1 also implies the identifiability of ${\mathrm{THR}}_{c(x)}\left(\pi \right)$ and ${\mathrm{THQ}}_{c(x)}\left(\pi \right)$ for any given policy π according to equations ${\mathrm{THR}}_{c(x)}\left(\pi \right)=E\left[{\mathrm{THR}}_{c(x)}\left(X\right)\pi \left(X\right)\right]$ and ${\mathrm{THQ}}_{c(x)}\left(\pi \right)=E\left[THQ_{c(x)}\left(X\right)\pi \left(X\right)\right]$.

**Proof of Theorem A1.** First, we prove (i). When σ(x,1)≠σ(x,0), the argument in Appendix A shows that, under Assumptions 1 and 2, $Y^{1}-Y^{0}|X=x∼N\left(\mu \left(x,1\right)-\mu \left(x,0\right),{\left(\sigma (x,1)-\sigma (x,0)\right)}^{2}\right)$. Moreover, μ(x,t) and σ(x,t) are identifiable for t=0,1. Therefore, both ${\mathrm{THR}}_{c(x)}\left(x\right)$ and ${\mathrm{THQ}}_{c(x)}\left(x\right)$ are identifiable.

$$\begin{array}{c} {\mathrm{THR}}_{c(x)}=P\left(Y^{1}-Y^{0}<c\left(x\right)|X=x\right)=\Phi \left(\frac{c(x)-(\mu (x,1)-\mu (x,0))}{\left|\sigma (x,1)-\sigma (x,0)\right|}\right). \end{array}$$

Let Z∼N(0,1), and denote $a=\mu \left(x,1\right)-\mu \left(x,0\right),\;b=\left|\sigma (x,1)-\sigma (x,0)\right|>0$. Then,

$$\begin{array}{cc} & {\mathrm{THQ}}_{c(x)}=E\left[\left(Y^{0}-Y^{1}+c\left(x\right)\right)I_{\left\{Y^{1}-Y^{0}<c\left(x\right)\right\}}|X=x\right]\\ = & -E[\left(Y^{1}-Y^{0}-c\left(x\right)\right)I_{\left\{Y^{1}-Y^{0}-c\left(x\right)<0\right\}}|X=x]\\ = & -\int_{-\infty }^{0}rf_{Y^{1}-Y^{0}-c\left(x\right)|X=x}\left(r\right)\;dr\\ = & -\int_{-\infty }^{0}r\frac{1}{\sqrt{2\pi }b}\exp\left\{-\frac{{\left\{r-a+c\left(x\right)\right\}}^{2}}{2b^{2}}\right\}\;dr\\ = & -\int_{-\infty }^{\frac{c(x)-a}{b}}\left(a-c\left(x\right)+bz\right)\frac{1}{\sqrt{2\pi }}\exp\left\{-\frac{z^{2}}{2}\right\}\;dz\\ = & \left(c(x)-a\right)\Phi \left(\frac{c(x)-a}{b}\right)+\frac{b}{\sqrt{2\pi }}\exp\left\{-\frac{{\left(c\left(x\right)-a\right)}^{2}}{2b^{2}}\right\}\\ = & \left(c(x)+\mu (x,0)-\mu (x,1)\right)\Phi \left(\frac{c(x)+\mu (x,0)-\mu (x,1)}{\left|\sigma (x,1)-\sigma (x,0)\right|}\right)\\ & +\frac{\left|\sigma (x,1)-\sigma (x,0)\right|}{\sqrt{2\pi }}\exp\left\{-\frac{{\left(c\left(x\right)+\mu \left(x,0\right)-\mu \left(x,1\right)\right)}^{2}}{2{\left(\sigma (x,1)-\sigma (x,0)\right)}^{2}}\right\}. \end{array}$$

This completes the proof of (i). Next, we prove (ii). Similarly, when σ(x,1)=σ(x,0), $Y^{1}-Y^{0}|X=x$ reduces to μ(x,1)−μ(x,0), according to the preceding proof, which implies that $P\left(Y^{1}-Y^{0}|X=x\right)$ can be identified under Assumption 1. Then we consider the identification of ${\mathrm{THR}}_{c(x)}\left(x\right)$ and ${\mathrm{THQ}}_{c(x)}\left(x\right)$.

$$\begin{array}{cc} {\mathrm{THR}}_{c(x)}\left(x\right)= & P\left(Y^{1}-Y^{0}<c\left(x\right)|X=x\right)\\ = & \left\{\begin{array}{cc} 0, & \;\mathrm{if}\;\;\mu (x,1)-\mu (x,0)⩾c(x)\\ 1, & \;\mathrm{if}\;\;\mu (x,1)-\mu (x,0)<c(x), \end{array}\right. \end{array}$$

$$\begin{array}{cc} \; & {\mathrm{THQ}}_{c(x)}\left(x\right)=E\left[\left(Y^{0}-Y^{1}+c\left(x\right)\right)I_{\left\{Y^{1}-Y^{0}<c\left(x\right)\right\}}|X=x\right]\\ = & -E[\left(Y^{1}-Y^{0}-c\left(x\right)\right)I_{\left\{Y^{1}-Y^{0}-c\left(x\right)<0\right\}}|X=x]\\ = & \left\{\begin{array}{cc} 0, & \;\mathrm{if}\;\;\mu (x,1)-\mu (x,0)⩾c(x)\\ \mu (x,0)-\mu (x,1)+c(x), & \;\mathrm{if}\;\;\mu (x,1)-\mu (x,0)<c(x). \end{array}\right. \end{array}$$

This completes the proof of (ii). □

Based on these idenifiability results, we can use the same methods in Section 4 to estimate ${\mathrm{THR}}_{c(x)}\left(\pi \right)$ and ${\mathrm{THQ}}_{c(x)}\left(\pi \right)$ for a given policy π, and learn a policy under the corresponding harm constraints. In summary, our results can be extended to settings in which activating the treatment incurs a cost c(x).

## Appendix E. Additional Experiments

**Performance under harm quantity constraints (RQ3).** Both harm rate and harm quantity can be calculated using Theorem 1 after estimation. Similar to imposing a constraint on the harm rate, we can also impose a constraint on the harm quantity. The optimization problem is then formulated as $\max_{\pi \in \Pi }\hat{R}\left(\pi \right),\mathrm{subject}\;\mathrm{to}\;\hat{\mathrm{THQ}}\left(\pi \right)⩽\delta_{q}$, where $\delta_{q}$ denotes the threshold for the harm quantity. The results across different threshold values are presented in Table A1, showing similar patterns to those in Table 1. We also conduct additional experiments for controlling both harm rate and harm quantity simultaneously in Appendix E.

**Table A1:** Performance under different harm quantity thresholds.

Ihdp	ΔWelfare	Reward	True Harm Rate	True Harm Quantity
Naive	48.329 ± 0.000	62.428 ± 0.000	0.484 ± 0.000	1.953 ± 0.000
CATE-based	1036.835 ± 2.414	1050.934 ± 2.414	0.138 ± 0.001	0.320 ± 0.004
Quantity (δ=0.03)	821.028 ± 78.425	835.127 ± 78.425	0.0422 ± 0.0034	0.0970 ± 0.0062
Quantity (δ=0.04)	858.436 ± 65.091	872.535 ± 65.091	0.0475 ± 0.0025	0.1102 ± 0.0035
Quantity (δ=0.05)	887.577 ± 54.048	901.676 ± 54.048	0.0538 ± 0.0023	0.1276 ± 0.0041
**Twins**	Δ **Welfare**	**Reward**	**True Harm Rate**	**True Harm Quantity**
Naive	−315.562 ± 0.000	−28.272 ± 0.000	0.5083 ± 0.0000	2.3258 ± 0.0000
CATE-based	10,227.300 ± 5.649	10,514.590 ± 5.649	0.0920 ± 0.0034	0.1675 ± 0.0077
Quantity (δ=0.040)	9853.759 ± 105.234	10,141.049 ± 105.234	0.0672 ± 0.0034	0.1196 ± 0.0060
Quantity (δ=0.045)	9890.298 ± 166.867	10,177.588 ± 166.867	0.0705 ± 0.0047	0.1263 ± 0.0076
Quantity (δ=0.050)	9991.342 ± 131.098	10,278.632 ± 131.098	0.0753 ± 0.0028	0.1354 ± 0.0045

The results under joint constraints on the harm rate and harm quantity are shown below in Table A2.

**Table A2:** Both rate and quantity.

Ihdp	ΔWelfare	Reward	True Harm Rate	True Harm Quantity
Naive	48.329 ± 0.000	62.428 ± 0.000	0.484 ± 0.000	1.953 ± 0.000
CATE-based	1036.835 ± 2.414	1050.934 ± 2.414	0.1378 ± 0.0011	0.3201 ±0.0039
$\delta_{r}=0.03,\delta_{q}=0.07$	949.563 ± 47.187	963.662 ± 47.187	0.059 ± 0.002	0.143 ± 0.007
$\delta_{r}=0.04,\delta_{q}=0.08$	958.354 ± 40.737	972.453 ± 40.737	0.061 ± 0.002	0.146 ± 0.008
$\delta_{r}=0.05,\delta_{q}=0.09$	982.042 ± 34.509	996.141 ± 34.510	0.067 ± 0.002	0.160 ± 0.006
**Twins**	Δ **Welfare**	**Reward**	**True Harm Rate**	**True Harm Quantity**
Naive	−315.562 ± 0.000	−28.272 ± 0.000	0.5083 ± 0.0000	2.3258 ± 0.0000
CATE-based	10,227.300 ± 5.649	10,514.590 ± 5.649	0.0920 ± 0.0034	0.1675 ± 0.0077
$\delta_{r}=0.03,\delta_{q}=0.10$	6642.551 ± 785.377	6929.841 ± 785.377	0.0559 ± 0.0013	0.1000 ± 0.0000
$\delta_{r}=0.05,\delta_{q}=0.12$	7513.270 ± 746.687	7800.560 ± 746.687	0.0663 ± 0.0013	0.1200 ± 0.0000
$\delta_{r}=0.07,\delta_{q}=0.14$	8565.449 ± 403.353	8852.739 ± 403.353	0.0769 ± 0.0007	0.1400 ± 0.0000

**Scale-Model Specification and THR Constraint Calibration (RQ4).** We add a specification experiment involving three nonlinear scale data-generating mechanisms: an interaction specification, a local-quadratic specification, and a threshold specification. For each mechanism, we compare the correctly specified and misspecified cases. Specifically, the three scale features are

$$\begin{array}{cc} f_{\mathrm{int}}\left(X\right) & =\;|X_{1}\left|+0.5\right|X_{2}X_{3}|,\\ f_{\mathrm{quad}}\left(X\right) & =\;|X_{1}|+0.5X_{2}^{2},\\ f_{\mathrm{thr}}\left(X\right) & =\;|X_{1}\left|+0.75I(\right|X_{2}\left|>1\right), \end{array}$$

and $\sigma_{a}\left(X\right)=\alpha_{a}+\beta_{a}f\left(X\right)$. We use $\left(\alpha_{0},\alpha_{1}\right)=\left(0.1,0.6\right)$ and $\left(\beta_{0},\beta_{1}\right)=\left(0.2,1.4\right)$ for the interaction mechanism; for the local-quadratic and threshold mechanisms, we use $\left(\beta_{0},\beta_{1}\right)=\left(0.2,1.2\right)$. The results over B=100 runs are reported in Table A3.

Across all three data-generating mechanisms, correctly specifying case substantially increases the frequency with which the estimated constraint is satisfied. Specifically, the estimated satisfaction counts increase from 82/100, 80/100, and 85/100 under misspecification to 98/100, 98/100, and 95/100 under correct specification. We emphasize, however, that these findings should not be interpreted as guaranteeing actual control of true harm, because finite-sample errors in $\hat{\mu }$ and $\hat{\sigma }$ continue to propagate into the learned policy.

**Table A3:** Calibration of the THR constraint on IHDP over B=100 repeated policy-training runs.

Setting	Estimated THR	True THR	Estimated Constraint Satisfaction	True-Harm Constraint Satisfaction	Mean Excess
Interaction (misspecified)	0.0267±0.0040	0.0341±0.0050	82/100	16/100	0.0046
Interaction (correct)	0.0293±0.0004	0.0413±0.0014	98/100	0/100	0.0113
Local-quadratic (misspecified)	0.0267±0.0035	0.0402±0.0046	80/100	0/100	0.0102
Local-quadratic (correct)	0.0291±0.0007	0.0437±0.0024	98/100	0/100	0.0137
Threshold (misspecified)	0.0270±0.0031	0.0486±0.0042	85/100	0/100	0.0186
Threshold (correct)	0.0294±0.0005	0.0523±0.0028	95/100	0/100	0.0223

## Appendix F. Implementation Details

Experiments were run on a MacBook Pro (Apple Inc., Cupertino, CA, USA) with an Apple M3 Pro chip, 18 GB RAM, and macOS 14.3. We used Python 3.9.6 and standard scientific computing libraries.

In the estimation phase, the outcome regression functions $\hat{\mu }\left(0\right)$, $\hat{\mu }\left(1\right)$ are modeled using an MLP in step 1, while the propensity score is estimated via logistic regression. In step 2, $\hat{\sigma }\left(0\right)$, $\hat{\sigma }\left(1\right)$ are modeled using an MLP under the MLE-based approach, and the regression-based method employs a linear regression model. For the harm-constrained policy learning procedure, we also use the MLP as the policy model and Adam as the optimizer. Table A4 presents the hyperparameter space for estimation and policy learning in both datasets.

**Table A4:** Implementation Details.

Hyperparameter	$\hat{\mu }$		$\hat{\sigma }$		Policy Learning	
	IHDP	TWINS	IHDP	TWINS	IHDP	TWINS
Learning rate	0.001	0.001	0.001	0.001	0.001	0.001
Batch size	20	20	20	20	20	64
Architecture (hidden layers)	[25,12]	[17,8]	[25,12]	[17,8]	[25,12]	[17,8]
Optimizer	Adam	Adam	Adam	Adam	Adam	Adam
Early stopping patience	20	20	20	20	20	20
Activation function (all layers)	ReLU	ReLU	ReLU	ReLU	ReLU	ReLU

## References

[1] Imai K., Strauss A. (2011). Estimation of heterogeneous treatment effects from randomized experiments, with application to the optimal planning of the get-out-the-vote campaign. Political Anal..

[2] Kaur P., Polyzou A., Karypis G. (2019). Causal inference in higher education: Building better curriculums. Proceedings of the 6th ACM Conference on Learning @ Scale (L@S 2019).

[3] Meid A.D., Ruff C., Wirbka L., Stoll F., Seidling H.M., Groll A., Haefeli W.E. (2020). Using the causal inference framework to support individualized drug treatment decisions based on observational healthcare data. Clin. Epidemiol..

[4] Chipman H.A., George E.I., McCulloch R.E. (2010). BART: Bayesian additive regression trees. Ann. Appl. Stat..

[5] Wager S., Athey S. (2018). Estimation and inference of heterogeneous treatment effects using random forests. J. Am. Stat. Assoc..

[6] Wang L., Zhou Y., Song R., Sherwood B. (2018). Quantile-optimal treatment regimes. J. Am. Stat. Assoc..

[7] Floridi L. (2019). Establishing the rules for building trustworthy AI. Nat. Mach. Intell..

[8] Kaur D., Uslu S., Rittichier K.J., Durresi A. (2022). Trustworthy artificial intelligence: A review. ACM Comput. Surv..

[9] Thiebes S., Lins S., Sunyaev A. (2021). Trustworthy artificial intelligence. Electron. Mark..

[10] Wu P., Shen Z., Xie F., Wang Z., Liu C., Zeng Y. Policy learning for balancing short-term and long-term rewards. Proceedings of the 41st International Conference on Machine Learning (ICML 2024).

[11] Yang Q., Liu X., Zeng Y., Guo R., Liu Y., Wu P. (2024). Learning the optimal policy for balancing short-term and long-term rewards. Proceedings of the Advances in Neural Information Processing Systems (NeurIPS 2024).

[12] Richens J., Beard R., Thompson D.H. (2022). Counterfactual harm. Proceedings of the Advances in Neural Information Processing Systems (NeurIPS 2022).

[13] Asimov I. (2004). I, Robot.

[14] Morath J.M., Turnbull J.E. (2005). To Do No Harm: Ensuring Patient Safety in Health Care Organizations.

[15] Sokol D.K. (2013). “First do no harm” revisited. BMJ.

[16] Kallus N. (2022). What’s the harm? Sharp bounds on the fraction negatively affected by treatment. Proceedings of the Advances in Neural Information Processing Systems (NeurIPS 2022).

[17] Lei L., Candès E.J. (2021). Conformal inference of counterfactuals and individual treatment effects. J. R. Stat. Soc. Ser. B Stat. Methodol..

[18] Li H., Zheng C., Cao Y., Geng Z., Liu Y., Wu P. Trustworthy policy learning under the counterfactual no-harm criterion. Proceedings of the 40th International Conference on Machine Learning (ICML 2023).

[19] Imbens G.W., Rubin D.B. (2015). Causal Inference for Statistics, Social, and Biomedical Sciences: An Introduction.

[20] Splawa-Neyman J., Dabrowska D.M., Speed T.P. (1990). On the application of probability theory to agricultural experiments. Essay on Principles. Section 9. Stat. Sci..

[21] Rubin D.B. (1974). Estimating causal effects of treatments in randomized and nonrandomized studies. J. Educ. Psychol..

[22] Holland P.W. (1986). Statistics and causal inference. J. Am. Stat. Assoc..

[23] Manski C.F. (2004). Statistical treatment rules for heterogeneous populations. Econometrica.

[24] Wu P., Ding P., Geng Z., Liu Y. (2026). Quantifying Individual Risk for Binary Outcomes. J. R. Stat. Soc. Ser. B Stat. Methodol..

[25] Shen C., Jeong J., Li X., Chen P.S., Buxton A. (2013). Treatment benefit and treatment harm rate to characterize heterogeneity in treatment effect. Biometrics.

[26] Bertsimas D., Kallus N., Weinstein A.M., Zhuo Y.D. (2016). Personalized diabetes management using electronic medical records. Diabetes Care.

[27] Murphy S.A. (2003). Optimal dynamic treatment regimes. J. R. Stat. Soc. Ser. B Stat. Methodol..

[28] Rosenbaum P.R., Rubin D.B. (1983). The central role of the propensity score in observational studies for causal effects. Biometrika.

[29] Hernán M.A., Robins J.M. (2020). Causal Inference: What If.

[30] Pearl J. (2009). Causality.

[31] Robins J.M., Rotnitzky A., Zhao L.P. (1994). Estimation of regression coefficients when some regressors are not always observed. J. Am. Stat. Assoc..

[32] Robins J.M., Rotnitzky A., Zhao L.P. (1995). Analysis of semiparametric regression models for repeated outcomes in the presence of missing data. J. Am. Stat. Assoc..

[33] Vaart A.W.v.d. (1998). Asymptotic Statistics.

[34] Vaart A.W.v.d., Wellner J.A. (2023). Weak Convergence and Empirical Processes.

[35] Hill J.L. (2011). Bayesian nonparametric modeling for causal inference. J. Comput. Graph. Stat..

[36] Almond D., Chay K.Y., Lee D.S. (2005). The costs of low birth weight. Q. J. Econ..

[37] Cheng L., Guo R., Liu H. (2021). Long-term effect estimation with surrogate representation. Proceedings of the 14th ACM International Conference on Web Search and Data Mining (WSDM 2021).

[38] Kitagawa T., Tetenov A. (2018). Who should be treated? Empirical welfare maximization methods for treatment choice. Econometrica.

[39] Athey S., Wager S. (2021). Policy learning with observational data. Econometrica.

[40] Ben-Michael E., Imai K., Jiang Z. (2023). Policy learning with asymmetric counterfactual utilities. arXiv.

[41] Chiappa S. (2019). Path-specific counterfactual fairness. Proceedings of the AAAI Conference on Artificial Intelligence (AAAI 2019).

[42] Kusner M.J., Loftus J., Russell C., Silva R. (2017). Counterfactual fairness. Proceedings of the Advances in Neural Information Processing Systems (NIPS 2017).

[43] Nabi R., Shpitser I. (2018). Fair inference on outcomes. Proceedings of the AAAI Conference on Artificial Intelligence (AAAI 2018).

[44] Yang Q., Chaoge J., Li J., Wu P. (2026). Counterfactual Harm-Aware Learning of Individualized Decisions. Neurocomputing.

[45] Zhang Z., Wang C., Nie L., Soon G. (2013). Assessing the heterogeneity of treatment effects via potential outcomes of individual patients. J. R. Stat. Soc. Ser. C (Appl. Stat.).

[46] Huang Y., Gilbert P.B., Janes H. (2012). Assessing treatment-selection markers using a potential outcomes framework. Biometrics.

[47] Yin Y., Cai Z., Zhou X.H. (2018). Using secondary outcome to sharpen bounds for treatment harm rate in characterizing heterogeneity. Biom. J..

[48] Sarvet A.L., Stensrud M.J. (2023). Perspective on ‘Harm’ in Personalized Medicine. Am. J. Epidemiol..

[49] Mueller S., Pearl J. (2023). Personalized Decision Making—A Conceptual Introduction. J. Causal Inference.

[50] Wu P., Mao X. (2026). The Promises of Multiple Experiments: Identifying Joint Distribution of Potential Outcomes. J. R. Stat. Soc. Ser. B Stat. Methodol..

[51] Shahn Z., Madigan D. (2025). Identification and Estimation of Joint Potential Outcome Distributions from a Single Study. arXiv.

[52] Beckers S., Chockler H., Halpern J.Y. (2022). A Quantitative Account of Harm. arXiv.

[53] Kosorok M.R. (2008). Introduction to Empirical Processes and Semiparametric Inference.

